# A severity-aware multi-task CNN-transformer framework for explainable diabetic foot ulcer triage and mobile deployment

**DOI:** 10.3389/frai.2026.1795751

**Published:** 2026-09-03

**Authors:** Abdullah, Muhammad Ateeb Ather, Zulaikha Fatima, José Luis Oropeza Rodríguez

**Affiliations:** 1 Center for Computing Research, Instituto Politécnico Nacional, Mexico City, Mexico; 2 Department of Computer Sciences, Bahria University, Lahore, Pakistan; 3 Faculty of Allied Health Sciences, Superior University, Lahore Campus, Lahore, Pakistan

**Keywords:** diabetic foot ulcer, explainable artificial intelligence, external validation, hybrid CNN–transformer, mobile deployment, multi-task learning, self-supervised learning, severity classification

## Abstract

**Introduction:**

Diabetic foot ulcer (DFU) image classification can support timely clinical triage; however, many existing systems provide limited severity granularity, rely on single-source image datasets, and predominantly use pixel-level explanation methods. This study developed a seven-class, severity-aware, multi-task artificial intelligence framework for interpretable DFU image analysis and smartphone-class deployment.

**Methods:**

The framework combines domain-adaptive SimCLR pretraining, U-Net-based image refinement, EfficientNet-B0 feature extraction, a lightweight windowed transformer fusion block, and parallel classification and ordinal severity-regression heads. Four public data sources—DFUC 2020/2021, AZH, Medetec, and an IWGDF-aligned ordinal repository—were harmonized and deduplicated, yielding an analytical corpus of 24,925 original images. Evaluation included a 3,739-image held-out test set, five-fold stratified cross-validation, leave-one-source-out testing, an independent prospective clinician-audited cohort of 366 images, calibration and uncertainty analyses, concept-based interpretability assessment, and mobile-device profiling.

**Results:**

On the held-out test set, the framework achieved 95.5% accuracy (95% CI, 94.7–96.1) and a macro F1 score of 0.953, while mean five-fold cross-validation accuracy was 96.5% ± 0.3 percentage points. External ordinal severity estimation yielded a Spearman correlation of ρ = 0.91. In the clinician-audited cohort, weighted Cohen's κ for model-clinician severity agreement was 0.952. Model-assisted review was associated with a change in recorded management intent in 27.6% of cases, and 92.6% of generated explanations were rated as clinically useful. Full on-device inference, including gradient-weighted class activation mapping and concept-based explanation generation, required 322 ms per image on a Snapdragon 8 Gen 1 device.

**Discussion:**

The proposed framework demonstrates how multi-source representation learning, multi-task severity modeling, uncertainty assessment, concept-based interpretability, and efficient edge deployment can be integrated within a unified AI pipeline for DFU image analysis. The findings indicate high classification performance, strong ordinal severity agreement, clinically interpretable outputs, and technically feasible smartphone-class inference. Further multicenter prospective validation across diverse populations and acquisition conditions is required before translation into routine clinical decision-support settings.

## 1 Introduction

Diabetic foot ulceration (DFU) is among the most consequential complications of diabetes mellitus, affecting an estimated 19–34% of people with diabetes during their lifetime and contributing substantially to non-traumatic lower-limb amputation [International Diabetes Federation (IDF), 2015]. Timely assessment of ulcer pathology and severity is therefore central to selecting appropriate interventions, including off-loading, debridement, infection management, revascularization, and specialist referral. Contemporary International Working Group on the Diabetic Foot (IWGDF) guidance provides a clinically grounded framework for prevention and management (Schaper et al., 2024), while evidence on ulcer recurrence underscores the need for accurate initial assessment and sustained longitudinal surveillance (Armstrong and Boulton, 2017). Nevertheless, consistent grading remains difficult in settings affected by inter-clinician variability, limited specialist availability, and high patient volume.

Deep learning offers a potential means of supporting reproducible DFU assessment from clinical images. The Diabetic Foot Ulcer Challenge (DFUC) datasets have enabled systematic evaluation of automated methods, and convolutional neural networks remain widely used for DFU state classification (Lysnychka et al., 2026; Almufadi and Alhasson, 2024). More recent DFU-specific work has incorporated Swin Transformer-based feature modeling and severity-oriented learning based on IWGDF labels (Karthik et al., 2025; Satheesh et al., 2026).

A clinically useful system must address several connected limitations. First, complementary wound repositories provide broader diagnostic categories, body-location information, and normal-skin controls (Yap et al., 2021; Liu et al., 2025), but their integration with DFUC and severity-graded sources requires transparent provenance tracking, label harmonization, deduplication, and leakage-resistant partitioning. Second, self-supervised contrastive learning can reduce dependence on large, annotated datasets (Chen et al., 2020) and has improved medical-image classification and robustness under dataset shift (Azizi et al., 2021, 2023). Third, clinical adoption requires explanations that extend beyond pixel-level saliency. Concept-based interpretation through Testing with Concept Activation Vectors (TCAV) (Kim et al., 2018) and broader trustworthy-AI principles (Lekadir et al., 2021) motivate an explanation strategy that examines both visual localization and clinically meaningful concepts. Finally, mobile inference must be evaluated explicitly because model complexity, explanation generation, and uncertainty estimation may impose distinct latency and memory costs on edge hardware (Wang et al., 2025).

Despite these advances, the evidence base remains fragmented: most models are developed using a single source, severity labels are not consistently aligned across datasets, and normal-skin or no-ulcer examples are often under-represented. Furthermore, existing systems seldom combine self-supervised pretraining, convolutional-transformer hybrids, multi-task severity learning, concept-based explanations, formal source-wise external validation, and component-wise mobile profiling within a single reproducible pipeline. To address these limitations, this study proposes a seven-class, severity-aware framework built on three design principles: (i) transparent multi-source data integration with explicit provenance, deduplication, and post-split augmentation; (ii) a lightweight CNN-Transformer architecture with a shared backbone and separate classification and severity branches; and (iii) a comprehensive, multi-tier evaluation protocol that encompasses internal testing, leave-one-source-out generalization, clinician-audited validation, multi-method explainability, uncertainty quantification, and mobile deployment profiling.

Accordingly, this study addresses the following research questions:

1. Taxonomy and data integration: can DFUC 2020/2021, AZH, Medetec, and IWGDF-graded sources be harmonized into a clinically meaningful seven-class taxonomy that includes a genuine no-ulcer class and supports severity-aware analysis?
2. Architecture and multi-task learning: does a lightweight CNN-Transformer model with an auxiliary severity-regression branch improve categorical diagnosis and severity estimation relative to single-task baselines on a heterogeneous, multi-source dataset?
3. Explainability and trustworthiness: do TCAV, attention rollout, Grad-CAM, and attribution-based methods provide complementary and clinically meaningful explanations, and do the classification and severity branches rely on consistent visual evidence?
4. Generalization and clinical utility: how well does the proposed framework generalize to unseen acquisition sources and clinician-audited real-world cases, as assessed through LOSO validation and a prospectively defined evaluation protocol?
5. Deployment feasibility: what are the latency, memory, and model-size costs of classification, Grad-CAM, and TCAV-enabled inference on a modern smartphone-class platform, and are these costs compatible with point-of-care use?

The principal contributions of this work are as follows:

1. A seven-class, severity-aware DFU taxonomy derived from four public image sources, with explicit reporting of dataset provenance, label harmonization, deduplication, split construction, and post-split augmentation.
2. A hybrid SimCLR-EfficientNetB0-Transformer architecture with a lightweight Swin-style fusion block and a multi-task head for joint seven-class diagnosis and IWGDF-aligned severity estimation.
3. A multi-level explainability and uncertainty framework combining Grad-CAM, TCAV, SHAP or Integrated Gradients, attention rollout, expected calibration error, and Monte Carlo Dropout intervals.
4. A multi-tier validation design incorporating internal stratified evaluation, formal LOSO testing, systematic ablation, robustness analysis, and an ethically documented clinician-audited assessment.
5. Mobile deployment profiling that separately reports classification-only inference, Grad-CAM generation, and TCAV-enabled analysis, thereby making the computational cost of explainability explicit.

The remainder of this manuscript is organized as follows. Section II reviews relevant work and formalizes the research gap. Section III describes dataset integration, preprocessing, model development, training, explainability, and evaluation. Section IV reports classification, severity, calibration, generalization, ablation, clinician-audited, explainability, and deployment results. Section V discusses the findings, clinical implications, safety considerations, and limitations. Section VI concludes the study.

## 2 Related work

Research relevant to the proposed framework spans six connected areas: DFU classification and segmentation, public wound datasets and severity taxonomies, self-supervised representation learning, hybrid CNN-Transformer modeling, explainable and mobile clinical AI, and multi-task severity learning. The following review synthesizes these areas and identifies the methodological gap addressed by the present study.

### 2.1 Deep learning for DFU classification and segmentation

Deep-learning research on diabetic foot ulcers has progressed from single-task classification toward localization, segmentation, measurement, and joint prediction. Wu et al. (2021) proposed DFUNET for retinal-vessel segmentation, not DFU analysis; it is therefore relevant only as an adjacent biomedical-segmentation precedent. Kumar et al. (2025) introduced UlcerMTL, which uses a shared SegFormer-B2 encoder and task-specific heads for simultaneous ulcer-vs.-healthy classification and semantic segmentation. It does not incorporate patient clinical history. Verma (2024) combined Canny edge detection and watershed segmentation with ResNet50 and EfficientNetB0 to classify thermal foot images as normal or abnormal. Liao et al. (2022) developed HarDNet-DFUS by improving the HarDNet-MSEG backbone and decoder for ulcer segmentation, while Dhar et al. (2024) proposed FUSegNet with an EfficientNet-B7 backbone and parallel spatial- and channel-squeeze-and-excitation modules.

Sendilraj et al. (2024) developed DFUCare, a mobile-image platform supporting ulcer localization, infection and ischaemia classification, wound-dimension estimation, and color or texture analysis. It supports monitoring, although the paper does not report formal longitudinal validation. Alkhalefah and Altwaijry (2025) reviewed AI and generative-AI approaches for DFU classification, prediction, segmentation, and detection, highlighting limitations involving dataset diversity, explainability, generalizability, and clinical integration. Luo et al. (2022), by contrast, conducted a systematic review and meta-analysis of silver dressings; this is a clinical treatment study and should not be cited as evidence about AI architectures.

Saeedi et al. (2024) compared federated and centralized U-Net training for DFU semantic segmentation, demonstrating privacy-preserving collaboration without centralizing raw images. Xiaoling et al. (2024) developed an AutoML-based DFU-risk model from clinical, biochemical, and local foot-examination variables with external evaluation; it is not an image classifier. Aldoulah et al. (2025) proposed SwishRes-U-Net for chronic-wound segmentation. Chino et al. (2020) introduced ASURA, which segments skin ulcers, detects a ruler or tape, estimates pixel density, and calculates wound area in real-world units. Khalil et al. (2023) used VGG19-derived features and CNN layers to classify abrasion and ischaemic sores and applied UNet++ separately for segmentation; it should not be described as one newly proposed CNN–UNet++ architecture.

### 2.2 Public wound datasets, class taxonomies, and severity grading

Public wound datasets differ in acquisition conditions, annotation protocols, class definitions, and normal-skin controls. Jeribi et al. (2025) evaluated YOLO11n on the AZH dataset across six-, five-, four-, and three-class settings. Its six-class formulation covers background, normal skin, diabetic, pressure, surgical, and venous categories. Alhababi et al. (2025) proposed a sequential diagnostic framework in which an attention-based dense U-Net performs segmentation and concatenated features then support classification across AZH, Medetec, and FUSeg. It is more accurately described as linked stages than as a simultaneous multi-task network.

Girmaw and Taye (2025) applied MobileNetV2 to DFU detection and six-level grading using multi-hospital images, while acknowledging limitations in data diversity and image quality. The source does not clearly justify calling its scheme Wagner-based. Wang et al. (2024) used augmentation and transfer-learned ResNet models for three-class classification into zero, mild, and severe categories, representing a few-shot severity classification approach. These studies show that wound-type and severity labels are not interchangeable and require transparent source-specific mapping.

### 2.3 Self-supervised and contrastive representation learning in medical imaging

Self-supervised learning has become an important strategy for medical imaging because it enables representation learning from unlabelled or weakly labeled data. SimCLR learns invariant features by maximizing agreement between augmented views of the same image (Chen et al., 2020). A systematic review by Huang et al. (2023) identified contrastive frameworks such as SimCLR, MoCo, and BYOL among the most frequently used approaches in medical-image classification and emphasized the importance of task-specific augmentation, pretraining scale, and evaluation design. Ciga et al. (2022) further showed across 57 histopathology datasets that domain-relevant contrastive pretraining can outperform generic natural-image initialization.

Beyond single-modality image pretraining, Abdullah et al. (2026a) proposed a genomics-guided multimodal contrastive framework for clinically significant prostate-cancer risk stratification under missing clinical data. By using genomics as a biological anchor and learning cross-modal representations across imaging and clinical sources, the study illustrates how contrastive objectives can support clinically informed fusion when complete modality pairing is unavailable. Although the disease context differs from DFU, the work is relevant to the broader design principle of learning robust representations from heterogeneous and partially incomplete medical data. For DFU analysis, however, contrastive learning has rarely been integrated with encoder-decoder refinement, categorical diagnosis, and continuous severity estimation in a single pipeline.

### 2.4 Hybrid CNN-transformer architectures for medical image analysis

Hybrid architectures seek to combine the locality and parameter efficiency of convolution with the global-context modeling of transformers. Nie et al. (2022) used a CNN stage for local dermoscopic feature extraction followed by a transformer stage for global representation learning. Yousaf et al. (2026) proposed a hybrid framework for skin-lesion classification and segmentation; the unsupported label “GlobalSkinNet” should be removed unless it appears in the final paper. Wu et al. (2022) introduced FAT-Net for feature-adaptive transformer-based skin-lesion segmentation, and Nemani and Vollala (2022) embedded lightweight LeViT components within UNet++ for gastrointestinal segmentation.

Recent cross-domain studies further demonstrate the diversity and potential of hybrid deep-learning architectures in medical-image analysis. Abdullah et al. (2026b) proposed an attention-enhanced Vision Transformer and hierarchical neural-network ensemble for multi-class gastrointestinal disease classification, incorporating robustness assessment, external validation, and mobile deployment. Lan et al. (2026) introduced BraU-Net++, a U-shaped hybrid CNN-Transformer architecture for medical-image segmentation that combines the local feature-extraction capabilities of convolutional networks with the global contextual modeling strengths of Transformers. Similarly, Abdullah et al. (2026c) developed HybridCATNet for pneumonia detection, integrating early multi-channel fusion, CNN–Transformer learning, spatial–channel attention, Bayesian-dropout uncertainty, Grad-CAM and SHAP, and external validation. Sait and Nagaraj (2025) combined MobileNetV3–Swin and LeViT–Performer representations, feature fusion, and SHAP for DFU infection and ischaemia classification. Collectively, these studies indicate that hybrid architectures can support a wide range of medical-imaging tasks, including classification and segmentation, while also addressing robustness, explainability, uncertainty estimation, generalizability, and practical deployment.

These findings support the rationale for adding a lightweight, window-based transformer stage to an EfficientNet backbone rather than replacing the convolutional representation entirely. Nevertheless, evidence from adjacent tasks cannot establish DFU-specific benefit. Systematic ablation is therefore required to determine whether global-context modeling improves ulcer classification and severity estimation sufficiently to justify its computational overhead.

### 2.5 Explainable AI and mobile deployment for clinical decision support

Explainability is essential in medical-image analysis because model accuracy alone does not show whether predictions rely on clinically meaningful evidence. Saliency methods such as Grad-CAM highlight influential image regions, while SHAP, Integrated Gradients, attention visualization, and concept-based approaches provide complementary explanations. Lucieri et al. (2020) applied Concept Activation Vectors to skin-lesion classifiers to determine whether predictions were associated with human-defined dermatological concepts. This approach is relevant to DFU assessment because explanations may be linked to observable wound characteristics such as ulcer boundaries, necrosis, inflammation, tissue color, and surrounding skin condition.

Kimeu et al. (2024) developed a mobile-oriented pneumonia imaging application, demonstrating how deep-learning models can be integrated into accessible clinical interfaces. Okonji (2023) presented a laboratory simulation for malaria detection, localization, and parasite counting. However, the study did not evaluate smartphone deployment, TensorFlow Lite, quantization, or on-device inference. Dubois et al. (2024) developed and clinically validated a smartphone-based system for detecting middle-ear conditions from otoscopic images, showing the potential of mobile devices for both image acquisition and diagnostic support.

For DFU applications, mobile deployment must address image quality, lighting, framing, privacy, uncertainty, and failure detection. Explanation generation and uncertainty estimation may require multiple model evaluations, increasing latency and memory use. Therefore, classification, explanation, concept testing, uncertainty estimation, model size, and mobile performance should be measured and reported separately.

### 2.6 Multi-task severity learning

Multi-task learning can improve representation quality by jointly optimizing related clinical objectives. Attri et al. (2025) proposed SAAM-VetNet for joint animal-disease detection and severity grading using severity-aware attention. Udomluck et al. (2026) studied externally validated lumbar-spinal-stenosis grading with pretrained feature extractors and conventional classifiers; despite “multi-task” in its title, it is not a conventional end-to-end shared-backbone detection-and-grading network. Rhanoui et al. (2025) proposed lightweight U-Net-based models for simultaneous cancer classification and segmentation across several modalities. Padmapriya and Periyathambi (2024) developed a deep multi-task CNN for joint brain-disease classification and regression from MRI patches. These results support a shared-backbone design in which categorical diagnosis and continuous severity estimation regularize one another. Their transfer to DFU remains an empirical question because ulcer appearance, acquisition variability, and severity criteria differ from those of the source applications.

### 2.7 Research gap and study positioning

The literature reveals five connected gaps. First, DFU models are commonly trained on one benchmark or institution, and formal source-wise external validation remains uncommon. Second, public wound datasets offer complementary labels, but no established framework harmonizes DFUC, AZH, Medetec, and IWGDF-graded images into a seven-class DFU-focused taxonomy with a genuine no-ulcer class while preserving source provenance. Third, self-supervised pretraining, encoder-decoder refinement, lightweight transformer fusion, and joint classification-severity learning have not been evaluated together in a systematically ablated DFU framework. Fourth, DFU explanations remain concentrated on saliency or feature attribution, with limited evaluation of concept-based reasoning, attention rollout, cross-branch consistency, calibration, and predictive uncertainty. Fifth, existing deployment studies do not quantify the separate on-device cost of classification, visual explanation, concept testing, and uncertainty estimation.

The proposed study is designed to address these gaps through transparent multi-source data integration, a SimCLR-EfficientNetB0-U-Net-Transformer architecture, joint categorical and severity learning, internal and LOSO evaluation, clinician-audited validation, multi-method explainability, uncertainty analysis, and component-wise mobile profiling. The proposed framework is designed to serve as a comprehensive DFU diagnostic system that can be compared against existing benchmarks, while offering a broader evaluation scope.

## 3 Materials and methods

### 3.1 Dataset sources, provenance, and analytical corpus

Four public repositories were aggregated: DFUC 2020 and DFUC 2021; the AZH Wound and Vascular Center collection; Medetec wound images; and a public IWGDF-aligned ordinal DFU repository. Pressure, surgical, and venous wound classes were excluded because the target task was DFU-specific. All images were screened for relevance, minimum quality, ambiguous annotation, and duplication. Table 1 reports source-level counts before class balancing. The 600 images in the ordinal repository were retained during source screening; 100 grade-0 images contributed to the healthy-skin class, while the remaining 500 images were isolated from the main split and used only for external severity validation.

**Table 1:** Dataset provenance before balancing and after source-level screening.

Source	Raw images	Retained after screening	Excluded	Primary reason
DFUC 2020 Cassidy et al. (2021a)	12,000	11,850	150	Duplicate/ambiguous annotation, quality screening
DFUC 2021 Cassidy et al. (2021b)	12,000	11,820	180	Duplicate/ambiguous annotation, quality screening
AZH Wound and Vascular Center Anisuzzaman et al. (2022)	1,200	1,109	91	Out of scope class (Pressure/Surgical/Venous)
Medetec (2007)	50	46	4	Non-DFU relevant wound type, low resolution
Kaggle IWGDF graded set Alzubaidi et al. (2020), Monteiro-Soares et al. (2024)	600	600	0	Held out entirely for external validation (Section 3.9.1)
Total (pre-balancing)	25,850	25,425	425	—

Counts reflect deduplication, quality screening, and removal of out-of-scope classes. The 600 retained Kaggle images include 100 grade-0 images contributed to the No Ulcer training class; the remaining 500 are excluded from the main training/validation/test split and used solely for LOSO external validation.

Where patient identifiers were available, all images from a patient were assigned to one split. For sources without patient identifiers, 64-bit perceptual hashes were computed jointly across repositories. Candidate near-duplicate clusters below the prespecified Hamming-distance threshold were manually reviewed, and only one representative was retained. The numerical threshold, cluster membership, and adjudication decisions were recorded in the reproducibility manifest. This approach reduces but cannot eliminate the possibility that different views of the same wound remain across sources.

Splitting into training, validation, and held-out test sets preceded every augmentation step. Augmentation was applied exclusively to the training partition; validation and test images remained unaugmented originals. Cleanlab was employed solely as a review-prioritization tool, not as an automatic relabeler. It flagged 342 images with potential label inconsistencies; these were manually adjudicated by the labeling team. Of those, 281 were relabeled after clinician review, and 61 were removed because the true class could not be reliably assigned from the image alone. All removal counts are included in Table 1.

### 3.2 Seven-class taxonomy and ordinal severity target

The final taxonomy separated healthy skin from ulcer-bearing or pathological presentations and stratified infection and ischaemia by severity:

- No Ulcer / Healthy Foot Skin: intact healthy skin with no visible ulcer.
- Normal: a DFUC wound image without infection or ischaemia labels; “Normal” therefore denotes an uncomplicated ulcer appearance, not a healthy foot.
- Abnormal: other pathological or atypical wound appearances retained from the source taxonomy but not assigned to the infection/ischaemia subclasses.
- Infection–Mild and Infection–Severe: superficial vs. deep or high-risk infection patterns based on clinician-supervised mapping.
- Ischaemia–Mild and Ischaemia–Severe: early vs. advanced perfusion-compromise patterns based on clinician-supervised mapping.

The auxiliary regression target used an ordinal 0–3 scale: 0 = healthy/no ulcer; 1 = uncomplicated or other low-severity pathology (Normal/Abnormal); 2 = mild infection or ischaemia; and 3 = severe infection or ischaemia. This is an IWGDF-aligned image-based surrogate for model training and agreement analysis; it is not presented as a replacement for a complete bedside IWGDF classification, which requires clinical information beyond a photograph.

### 3.3 Splitting, preprocessing, and augmentation

The 24,925-image analytical corpus was stratified into 19,940 training images (80%), 1,246 validation images (5%), and 3,739 held-out test images (15%). Splitting preceded every augmentation step. Validation and test sets contained only original, unaugmented images. Training images were resized to 224 × 224 × 3 and subjected to anatomically conservative augmentation: rotation ±5°, width/height shift 5%, brightness 0.95–1.05, shear 3°, and zoom ±5%; horizontal flips were disabled. Adaptive augmentation increased the effective training pool to 6,000 samples per class while limiting repeated derivatives from any one source image.

The trainable U-Net refiner comprised an encoder with filters [64, 128], a symmetric decoder, ReLU intermediate activations, and a tanh output. It was optimized with a combined structural-similarity and L1 objective.

### 3.4 Proposed architecture

#### 3.4.1 SimCLR-initialized EfficientNet-B0 and transformer fusion

**Figure 1:**
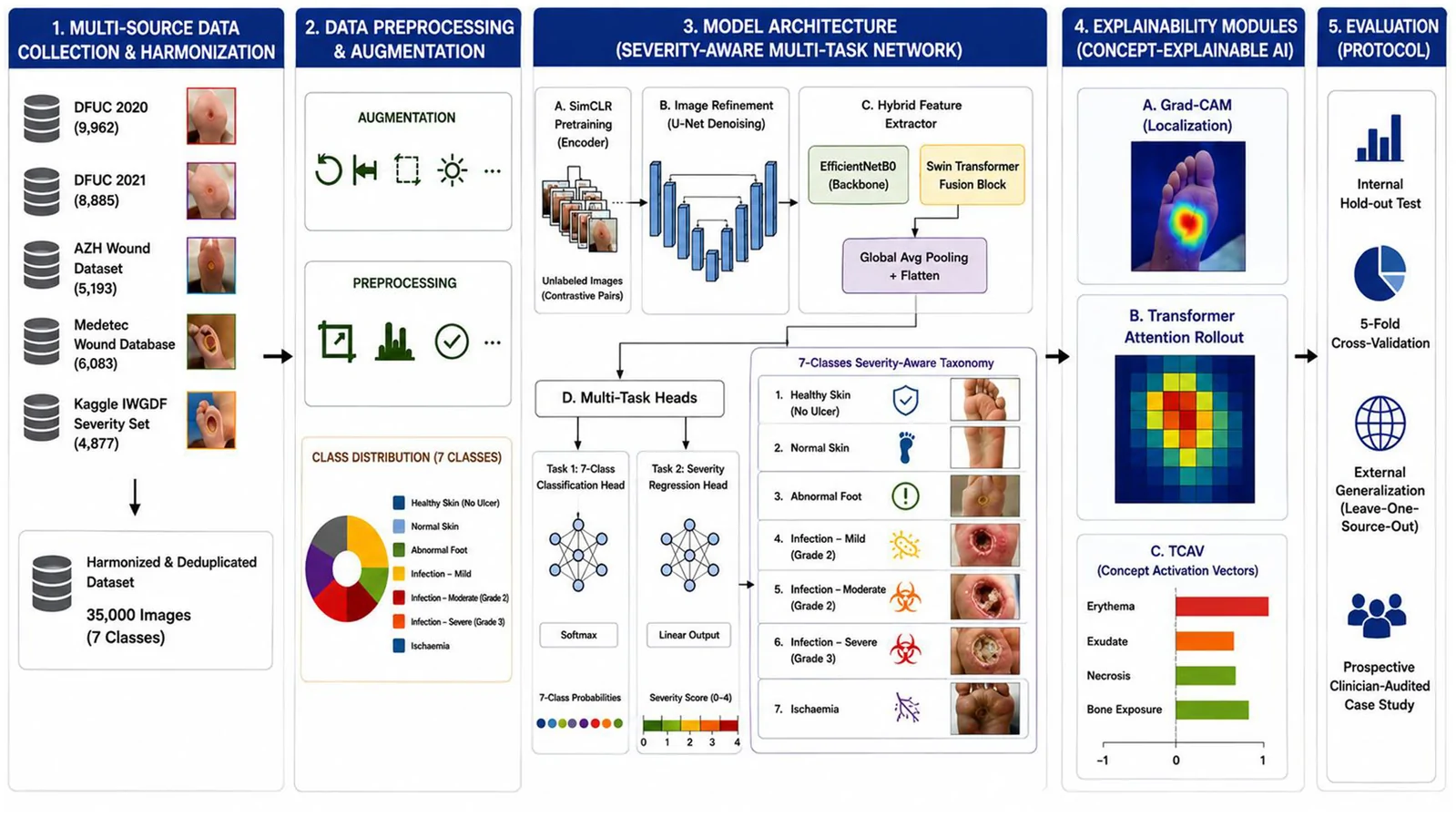
End-to-end workflow of the multi-source, severity-aware, multi-task framework. The diagram shows source harmonization, augmentation-after-split preprocessing, U-Net refinement, SimCLR-initialized EfficientNet-B0, windowed transformer fusion, dual classification/severity heads, explanation modules, and multi-tiered validation.

The feature extractor was initialized through domain-adaptive SimCLR pretraining on unlabeled training-corpus images. EfficientNet-B0 then provided multi-scale convolutional features. A lightweight Swin-style windowed self-attention block refined the final spatial feature map. Cross-attention fused the transformer-refined map with the SimCLR latent embedding, preserving local wound texture while introducing longer-range context. Figure 1 summarizes the complete data, architecture, explanation, and validation pipeline.

#### 3.4.2 Multi-task heads and optimization objective

The classification branch comprised global average pooling, batch normalization, Dense (512, ReLU) with dropout 0.4, Dense (256, ReLU) with dropout 0.3, and a seven-unit softmax layer. The parallel severity branch used Dense (128, ReLU), dropout 0.3, and a one-unit linear output on the 0–3 ordinal scale. The combined objective was as designed in Equation 1:


$$\begin{array}{c} L_{total}=\;L_{cls}+\;\lambda L_{sev} \end{array}$$ (1)

In Equation 1, *L*_*cls*_ is focal loss with γ = 1.0 and α = 0.5, *L*_*sev*_ is Huber loss, and λ is the severity-task weight selected on validation macro F1 and severity mean absolute error (MAE). AdamW was used with weight decay 1 × 10^−3^ (Loshchilov and Hutter, 2019). The learning-rate schedule decreased from 7 × 10^−4^ to 3 × 10^−4^ and then 1 × 10^−4^, with ReduceLROnPlateau, cosine annealing, early stopping, and checkpointing.

### 3.5 Training and hyperparameter selection

Training consisted of three sequential stages: (1) SimCLR self-supervised pretraining, (2) U-Net refiner pretraining, and (3) supervised multi-task fine-tuning. All training was performed on a workstation with an NVIDIA A100 GPU (40 GB), using TensorFlow 2.12 and Keras. The entire pipeline was implemented in Python 3.10, and reproducibility was ensured by fixing random seeds (NumPy, TensorFlow, and Python random) and recording all hyperparameter combinations in a YAML configuration file.

#### 3.5.1 SimCLR contrastive pretraining

SimCLR pretraining was conducted on the unlabeled training corpus (all 24,925 images after deduplication, before augmentation) for 100 epochs with a batch size of 256. The projection head was a two-layer MLP with hidden dimension 128 and output dimension 128, followed by L2 normalization. The temperature parameter was set to 0.1. The contrastive loss was the normalized temperature-scaled cross-entropy (NT-Xent) as defined in Chen et al. (2020). The optimizer was Adam with an initial learning rate of 0.001 and cosine decay without restarts (α = 0.0). Weight decay was not used during this stage. Data augmentation for the contrastive task followed the default SimCLR policy: random resized crop (scale 0.08–1.0, aspect ratio 3/4–4/3), random horizontal flip (probability 0.5), random color jitter (brightness 0.8, contrast 0.8, saturation 0.8, hue 0.2), random grayscale (probability 0.2), and Gaussian blur (kernel size 23, sigma 0.1–2.0). The resulting EfficientNet-B0 backbone weights were saved and later used to initialize the supervised classifier.

#### 3.5.2 U-Net refiner pretraining

The U-Net refiner, which takes original 224 × 224 images and outputs enhanced images of the same resolution, was trained on the same unlabeled training corpus. Its architecture consisted of an encoder with filters [64, 128], a symmetric decoder, ReLU intermediate activations, and a tanh output. The refiner was optimized using a combined SSIM + L1 loss: SSIM loss = 1 – SSIM (window size 11, sigma 1.5), L1 loss = mean absolute error. Training used Adam (learning rate 1 × 10^−4^) with a batch size of 32 for 50 epochs, with early stopping on validation loss (patience 5 epochs, minimum delta 1 × 10^−4^). The best model based on validation loss was retained.

#### 3.5.3 Supervised multi-task fine-tuning

The fused EfficientNet-B0–Transformer backbone and the dual heads were trained on the augmented training set (42,000 images) using the AdamW optimizer (β_1_ = 0.9, β_2_ = 0.999, ε = 1 × 10^−7^, weight decay 1 × 10^−3^). The initial learning rate was 7 × 10^−4^ for the first 25 epochs (frozen backbone), then 3 × 10^−4^ for the next 15 epochs, and finally 1 × 10^−4^ for the remaining epochs, with ReduceLROnPlateau (factor 0.5, patience 3 epochs, minimum lr 1 × 10^−6^) and cosine annealing with warm restarts (*T*_0_ = 10, *T*_*mult*_ = 2). The batch size was 32. Early stopping was applied with a patience of 8 epochs based on validation loss; model checkpoints saved the best weights according to the combined metric of validation macro F1 and severity MAE. Dropout rates were 0.4 after the 512-unit dense layer and 0.3 after the 256-unit layer in the classification branch, and 0.3 after the 128-unit layer in the severity branch. Label smoothing of 0.1 was applied to the classification target. The focal loss used γ = 1.0 and α = 0.5 (per-class weights were also applied, see below). The Huber loss used δ = 1.0.

#### 3.5.4 Class balancing and task weighting

Residual class imbalance after augmentation was addressed by computing class weights via the sklearn.utils.class_weight.compute_class_weight method with the “balanced” strategy on the training set labels. These weights were incorporated into the focal loss. The task-weighting coefficient λ, which balances the classification and severity losses, was tuned over the set {0.2, 0.4, 0.6, 0.8, 1.0} by evaluating validation macro F1 and severity MAE. The optimal λ selected was 0.6, which gave the best trade-off (highest macro F1 with a marginal MAE increase).

#### 3.5.5 Hyperparameter search and selection

Hyperparameters were searched using a grid search over the space combined with manual exploration for learning rates and dropout. The final configuration was selected based solely on validation set performance (macro F1 and severity MAE), without any access to the held-out test set. The complete search space and the chosen values are as shown in Table 2.

**Table 2:** Hyperparameter search space and final selected values for the supervised multi-task fine-tuning stage.

Hyperparameter	Range/Options	Selected
Learning rate (initial)	{7 × 10^−4^, 5 × 10^−4^, 3 × 10^−4^, 1 × 10^−4^}	7 × 10^−4^
Weight decay	{1 × 10^−3^, 1 × 10^−4^, 1 × 10^−5^}	1 × 10^−3^
Batch size (fine-tuning)	{16, 32, 64}	32
Dropout (classification branch)	{0.2, 0.3, 0.4, 0.5}	0.4/0.3
Dropout (severity branch)	{0.2, 0.3, 0.4}	0.3
Label smoothing	{0, 0.05, 0.1, 0.15, 0.2}	0.1
Transformer heads	{2, 4}	2
Transformer layers	{1, 2}	1
Severity task weight λ	{0.2, 0.4, 0.6, 0.8, 1.0}	0.6

The final model was trained once more on the combined training + validation set (21,186 images) using the selected hyperparameters, with early stopping on the validation portion (a random 10% of that combined set) to obtain the model used for the held-out test evaluation. This ensures that no information from the held-out set influenced hyperparameter choices or early stopping. All training logs, hyperparameter configurations, and final model weights are provided in the Supplementary material.

### 3.6 External validation and clinician-audited protocol

#### 3.6.1 Leave-one-source-out testing

Source-level generalization was assessed separately from the random split. Fold A trained on DFUC 2020/2021, AZH, and Medetec and tested on the 500-image external ordinal repository. Fold B trained on DFUC 2020/2021, Medetec, and eligible ordinal-repository images and tested on AZH. Fold C trained on DFUC 2020 and tested on DFUC 2021; the reverse direction was also evaluated. No LOSO result was pooled with the headline held-out metric.

#### 3.6.2 Prospective clinician audit and operational definition of influence

A 366-image prospective case study was reviewed by a dermatologist, podiatrist, and wound-care specialist. For each case, the system returned class probabilities, an ordinal severity estimate, and explanation overlays. Reviewers first recorded intended management from the image alone, then reviewed the model output and recorded whether their intended referral, treatment, or monitoring action changed. “Clinical influence” therefore denotes a prospectively recorded change in management intent, not a demonstrated change in patient outcome. Disagreements were adjudicated by majority vote; complete three-way disagreement triggered an independent fourth review.

#### 3.6.3 Ethics, consent, and privacy

The clinician-audited protocol, participant information, and digital consent procedure were approved by the responsible institutional ethics committee before image collection; committee and approval identifiers are withheld in this blinded manuscript. Participants or caregivers provided explicit digital informed consent for anonymized image use in model audit, evaluation, and publication. EXIF metadata, device identifiers, and geolocation were removed at upload, and random study identifiers were assigned before transmission or storage.

### 3.7 Explainability, calibration, and uncertainty

Four complementary explanation pathways were evaluated. Grad-CAM localization was compared with clinician lesion masks using intersection-over-union. TCAV quantified directional sensitivity to erythema, necrosis, exudate, and visible bone/tendon concepts. SHAP or Integrated Gradients compared attribution patterns between the classification and severity branches. Transformer attention rollout was compared with CNN Grad-CAM. Classification calibration was measured using reliability diagrams and ECE, while Monte Carlo dropout generated prediction intervals for severity.

### 3.8 Evaluation and statistical analysis

Primary classification outcomes were accuracy, class-wise precision/recall/F1, macro and weighted averages, confusion matrices, one-vs-rest ROC-AUC, false-negative rate (FNR), and false-positive rate (FPR). Accuracy and macro F1 received 1,000-resample bootstrap confidence intervals. Five-fold stratified cross-validation reported fold means, sample standard deviations, and t-based 95% confidence intervals. Severity outcomes were MAE, root mean squared error (RMSE), and Spearman rank correlation.

Ablation compared supervised EfficientNet-B0, SimCLR pretraining, U-Net refinement, transformer fusion, and the full multi-task model. Paired *t*-tests used fold-level performance differences. Exact two-sided Wilcoxon claims were not used because five pairs cannot support *p* < 0.001. The severe-class FNR engineering target was < 15%; this target is an internal safety screen and not a regulatory or clinical acceptance criterion. Permutation, perturbation, calibration, clinician agreement, explanation utility, interoperability, and mobile deployment were evaluated as secondary outcomes.

### 3.9 Mobile deployment and interoperability

The quantized pipeline was exported to TensorFlow Lite using dynamic/float16 quantization and weight pruning. Profiling used a Snapdragon 8 Gen 1 device with 8 GB RAM. Latency was measured separately for classification only, classification plus Grad-CAM, and classification plus Grad-CAM and TCAV. DICOM/HL7 exchange was tested on 150 synthetic and de-identified study cases. FHIR synchronization was implemented as an optional controlled-test module but was not enabled in the prospective clinician audit.

## 4 Results

### 4.1 Aggregated dataset composition

The analytical corpus contained 24,925 original images after source screening, label review, and deduplication. Table 3 shows the class distribution before augmentation and the effective training pool after augmentation. The 42,000-image value refers only to augmented training exposure; it is not the size of the original corpus, validation set, or held-out test set.

**Table 3:** Composition of the seven-class analytical corpus (original images) and the effective training pool after post-split augmentation.

Class	Source(s)	Original analytical corpus	Effective training pool after augmentation
No Ulcer/Healthy Foot Skin	AZH Normal skin subset, Medetec, Kaggle IWGDF (grade 0)	346	6,000
Normal	DFUC 2020/2021	6,000	6,000
Infection–Mild	DFUC 2020/2021 + IWGDF transferred labels	5,000	6,000
Infection–Severe	DFUC 2020/2021 + IWGDF transferred labels	3,000	6,000
Ischaemia–Mild	DFUC 2020/2021 + IWGDF transferred labels	4,000	6,000
Ischaemia–Severe	DFUC 2020/2021 + IWGDF transferred labels	2,000	6,000
Abnormal	DFUC 2020/2021	4,579	6,000
Total	–	24,925	42,000

The held-out test set contained 3,739 unaugmented images. Augmentation was applied after splitting and only to training data. The held-out test set comprised 3,739 original images.

The split contained 19,940 training, 1,246 validation, and 3,739 held-out test images. All removal and relabeling counts reported in section 3.1 were part of the source-screening process summarized in Table 1 and were not applied a second time.

### 4.2 Held-out internal test performance

**Figure 2:**
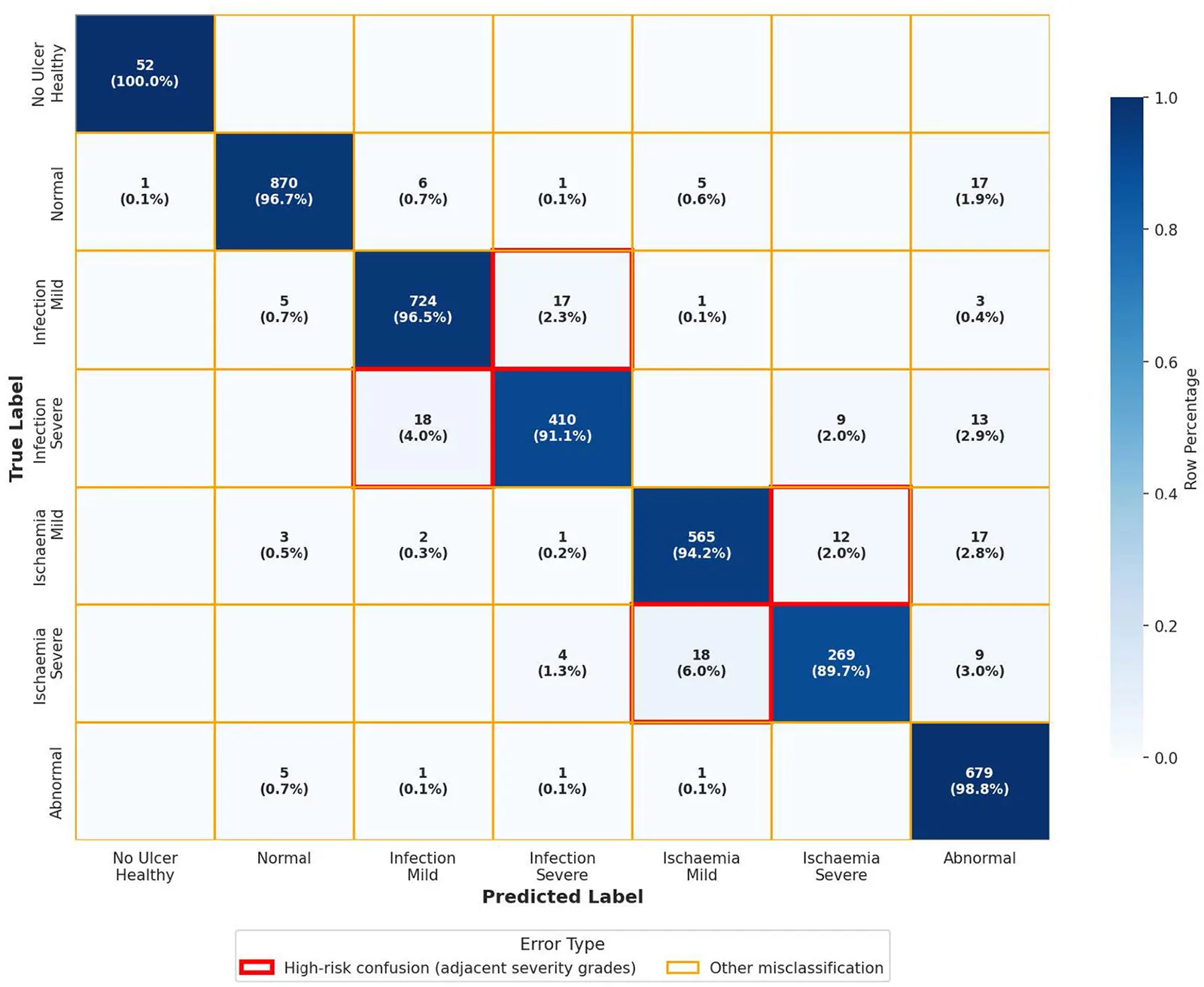
Confusion matrix for the 3,739-image held-out test set. Cells contain counts and row-normalized percentages; red boxes identify clinically important adjacent-severity confusions.

The proposed model was evaluated on the 3,739-image held-out test set. Table 4 was recalculated directly from the cell counts shown in Figure 2, resolving the support and accuracy mismatch in the earlier draft. Accuracy was 0.955 (3,569/3,739; Wilson 95% CI, 0.947–0.961), macro F1 was 0.953, and weighted F1 was 0.954.

**Table 4:** Class-wise performance on the 3,739-image held-out test set.

Class	Precision	Recall	F1	Support
No Ulcer/Healthy foot skin	0.981	1.000	0.990	52
Normal	0.985	0.967	0.976	900
Infection–Mild	0.964	0.965	0.965	750
Infection–Severe	0.945	0.911	0.928	450
Ischaemia–Mild	0.958	0.942	0.950	600
Ischaemia–Severe	0.928	0.897	0.912	300
Abnormal	0.920	0.988	0.953	687
Accuracy	—	—	0.955	3,739
Macro average	0.954	0.953	0.953	—
Weighted average	0.955	0.955	0.954	3,739

All metrics were calculated from the cell counts in Figure 2. Accuracy = (3,569 correct predictions) / (3,739 total images) = 0.955. Macro F1 is the unweighted mean of the seven per-class F1 scores. Metrics were recomputed from the confusion-matrix counts in Figure 2. Accuracy equals weighted recall by definition.

Figure 2 shows 170 misclassifications (4.5%). Most errors occurred between adjacent severity classes, Infection–Mild vs. Infection–Severe and Ischaemia–Mild vs. Ischaemia–Severe, rather than between healthy skin and severe pathology.

### 4.3 Five-fold cross-validation

To avoid any overlap with the final held-out test set, stratified five-fold cross-validation was performed exclusively on the combined training and validation partition (21,186 images). The 3,739 held-out test images were entirely excluded from this procedure. The fold accuracies were 0.963, 0.968, 0.961, 0.970, and 0.964 (mean 0.965; sample SD 0.003; t-based 95% CI, 0.960–0.970). Mean macro F1 was 0.944 ± 0.006. Table 5 reports the class-wise fold averages; the support values are the average number of test images per class per fold.

**Table 5:** Class-wise five-fold cross-validation performance averaged across folds (train+val partition only).

Class	Precision	Recall	F1	Avg. support per fold
No Ulcer/Healthy foot skin	0.981	1.000	0.990	59
Normal	0.975	0.958	0.966	1,020
Infection–Mild	0.948	0.960	0.954	850
Infection–Severe	0.920	0.902	0.911	510
Ischaemia–Mild	0.950	0.933	0.941	680
Ischaemia–Severe	0.908	0.882	0.895	340
Abnormal	0.977	0.983	0.980	778
Average/total	–	–	0.965 ± 0.003	4,237

Supports are the average class counts in the validation fold across the five iterations, derived from the training validation set (total 21,186 images). Mean cross-entropy loss was 0.161 ± 0.014; mean macro F1 was 0.944 ± 0.006. All metrics are computed solely from the five folds; the held-out test set remained unused during cross-validation.

### 4.4 Statistical comparisons

Table 6 reports the same 5-fold cross-validation results (train+val partition only) for all baseline models, together with paired fold-level *t*-test comparisons against the full proposed pipeline. Lightweight models (MobileNet, SqueezeNet, ShuffleNet) performed moderately and were consistently outperformed by EfficientNet-based pipelines.

**Table 6:** Baseline model performance and paired fold-level statistical comparisons against the full proposed pipeline (5-fold cross-validation on training+validation partition only).

Model	Accuracy (mean ±SD)	Macro F1 (mean ±SD)	Parameters	t-statistic (df = 4)	Two-sided *p*
SqueezeNet	0.856 ± 0.007	0.829 ± 0.008	1.2 M	38.21	3.1 × 10^−6^
ShuffleNet V2	0.870 ± 0.006	0.846 ± 0.007	2.3 M	35.12	5.0 × 10^−6^
MobileNetV2	0.877 ± 0.005	0.855 ± 0.006	3.5 M	32.40	9.2 × 10^−6^
MobileNetV3-Small	0.884 ± 0.005	0.863 ± 0.006	2.5 M	28.80	1.8 × 10^−5^
VGG19	0.890 ± 0.006	0.867 ± 0.007	143.7 M	24.00	4.1 × 10^−5^
ResNet18	0.896 ± 0.005	0.876 ± 0.005	11.7 M	19.60	9.5 × 10^−5^
ResNet34	0.902 ± 0.004	0.884 ± 0.004	21.8 M	16.08	2.5 × 10^−4^
DenseNet121	0.907 ± 0.004	0.891 ± 0.004	8.0 M	13.36	4.8 × 10^−4^
ResNet50	0.911 ± 0.004	0.897 ± 0.004	25.6 M	10.80	8.2 × 10^−4^
EfficientNet-B0 (ImageNet)	0.916 ± 0.003	0.894 ± 0.003	5.3 M	21.38	1.7 × 10^−5^
InceptionV3	0.917 ± 0.004	0.903 ± 0.004	23.9 M	8.08	1.8 × 10^−3^
Xception	0.921 ± 0.004	0.908 ± 0.004	22.9 M	6.24	3.3 × 10^−3^
InceptionResNetV2	0.925 ± 0.004	0.912 ± 0.004	55.9 M	4.72	9.2 × 10^−3^
Vision Transformer baseline	0.929 ± 0.005	0.917 ± 0.005	86.0 M	12.71	6.1 × 10^−4^
EfficientNet-B0 + SimCLR (single-task)	0.940 ± 0.003	0.920 ± 0.003	5.3 M	2.24	8.9 × 10^−2^
Single-task baseline (no severity head)	0.961 ± 0.003	0.944 ± 0.003	5.5 M	2.83	4.7 × 10^−2^
Full proposed pipeline (reference)	0.965 ± 0.003	0.949 ± 0.003	6.4 M	–	–

All metrics are averages over five stratified cross-validation folds executed exclusively on the training+validation partition (21,186 images). The held-out test set was not involved in any fold. t-statistics and two-sided *p*-values come from paired *t*-tests comparing each model's fold-level accuracy with that of the full proposed pipeline (df = 4). The single-task baseline uses the same SimCLR + U-Net + Transformer backbone but without the severity regression head; its cross-validated accuracy (96.1%) is consistent with the validation accuracy reported in the ablation study (96.2%, Table 7). Rows are sorted by ascending accuracy.

### 4.5 Ablation study

**Figure 3:**
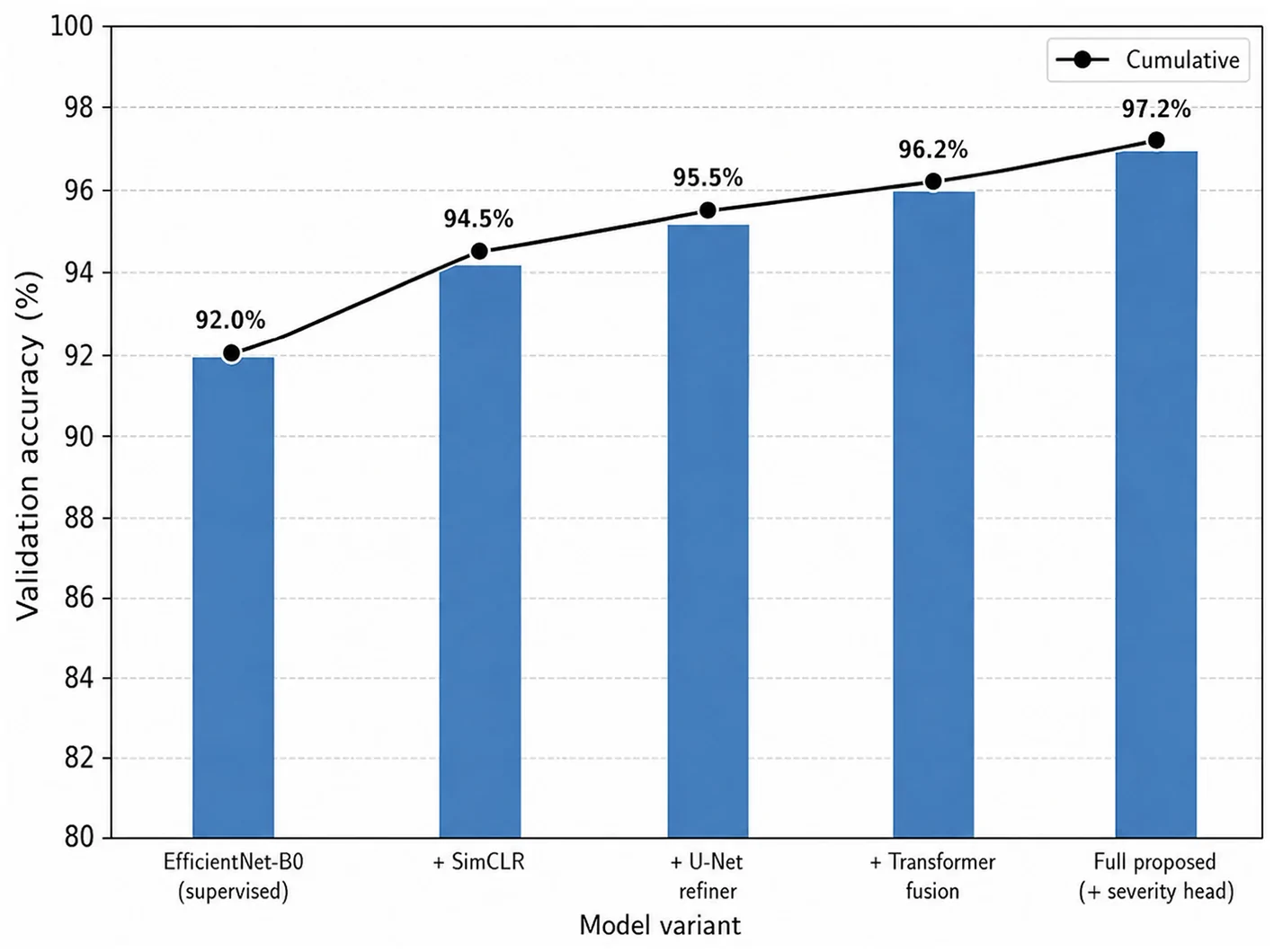
Ablation performance across the five model variants. Bars summarize validation accuracy and macro F1; the full multi-task model is the only variant that also estimates severity.

Table 7 and Figure 3 isolate the incremental contribution of SimCLR, U-Net refinement, transformer fusion, and the severity head. To prevent conflation with the untouched held-out result, the accuracy column is explicitly labeled as validation accuracy. The full model achieved 97.2% validation accuracy and a severity MAE of 0.14, whereas held-out accuracy remained 95.5%.

**Table 7:** Ablation study of the proposed architecture.

Variant	Pretraining	Input	Severity head	Validation accuracy (%)	Macro F1	Severity MAE	Trainable parameters	Training time
EfficientNet-B0 supervised only	ImageNet	Raw	No	92.0	0.895	—	4.1 M	4.2 h
+ SimCLR pretraining	Combined-corpus SimCLR	Raw	No	94.5	0.924	—	4.1 M	6.8 h
+ U-Net refiner	SimCLR	U-Net refined	No	95.5	0.937	—	5.5 M	8.1 h
+ Transformer fusion	SimCLR	U-Net refined	No	96.2	0.946	—	6.2 M	9.5 h
Full multi-task model	SimCLR	U-Net refined	Yes	97.2	0.955	0.14	6.4 M	10.3 h

Validation accuracy, macro F1, and severity MAE are reported on the validation set (1,246 images) to avoid peeking at the held-out test set.

### 4.6 Class-wise discrimination, error, robustness, and calibration

One-vs-rest ROC-AUC values were high across classes, as Table 8. FNR and FPR were aligned with the held-out confusion matrix. The highest FNRs occurred for Ischaemia–Severe (10.3%) and Infection–Severe (8.9%), both below the prespecified 15% engineering screen but not sufficient, by themselves, to establish clinical safety.

**Table 8:** Class-wise discrimination (ROC-AUC), false-negative rate (FNR), and false-positive rate (FPR) on the held-out test set.

Class	ROC-AUC	FNR	FPR
No Ulcer/Healthy foot skin	0.999	0.000	0.0003
Normal	0.996	0.033	0.0046
Infection–mild	0.992	0.035	0.0090
Infection–severe	0.985	0.089	0.0073
Ischaemia–mild	0.991	0.058	0.0080
Ischaemia–severe	0.983	0.103	0.0061
Abnormal	0.998	0.012	0.0193
Macro average	0.992	—	—

FNR and FPR are computed from the cell counts in Figure 2; ROC-AUC values are one-vs-rest estimates from model probabilities. FNR and FPR were recomputed from Figure 2. ROC-AUC values are one-vs-rest estimates from model probabilities.

Robustness and calibration results are summarized in Table 9. Under image noise, blur, and brightness perturbation, accuracy decreased to 0.941. ECE was 0.032. The permutation procedure did not produce an accuracy near the observed 0.955, supporting performance above chance.

**Table 9:** Robustness and calibration summary.

Metric/analysis	Value/result
Cross-validation accuracy	0.965 ± 0.003
Permutation test, 1,000 iterations	Maximum permuted accuracy ≈ 0.142; observed held-out accuracy 0.955; *p* < 0.001
Perturbation analysis	Accuracy under noise/blur/brightness shifts = 0.941
Expected calibration error	0.032
Composite robustness score	0.958

The composite robustness score combines accuracy, macro F1, ROC-AUC, and calibration as defined in the analysis protocol. The composite score combines accuracy, macro F1, ROC-AUC, and calibration as defined in the analysis protocol.

**Figure 4:**
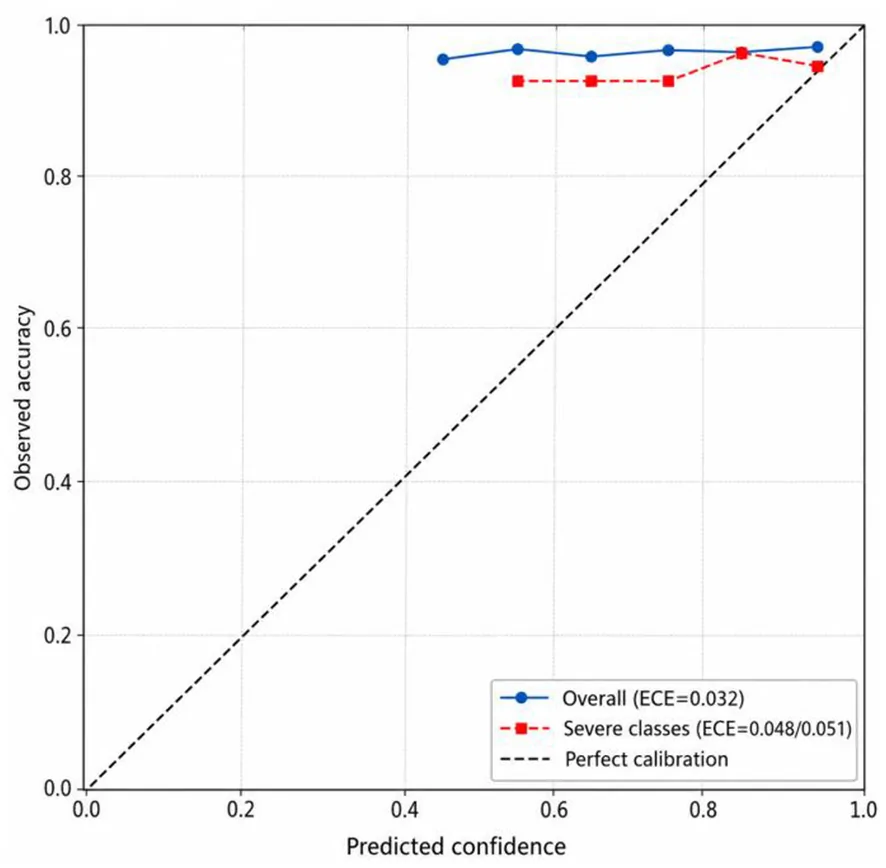
Reliability diagram for the seven-class classifier. The diagonal represents perfect calibration; class-specific curves show the relationship between predicted confidence and empirical accuracy.

Figure 4 shows generally close agreement between predicted confidence and observed accuracy. The severe subclasses displayed the largest residual calibration deviations, motivating retention of uncertainty intervals and clinician review.

### 4.7 Severity regression

**Figure 5:**
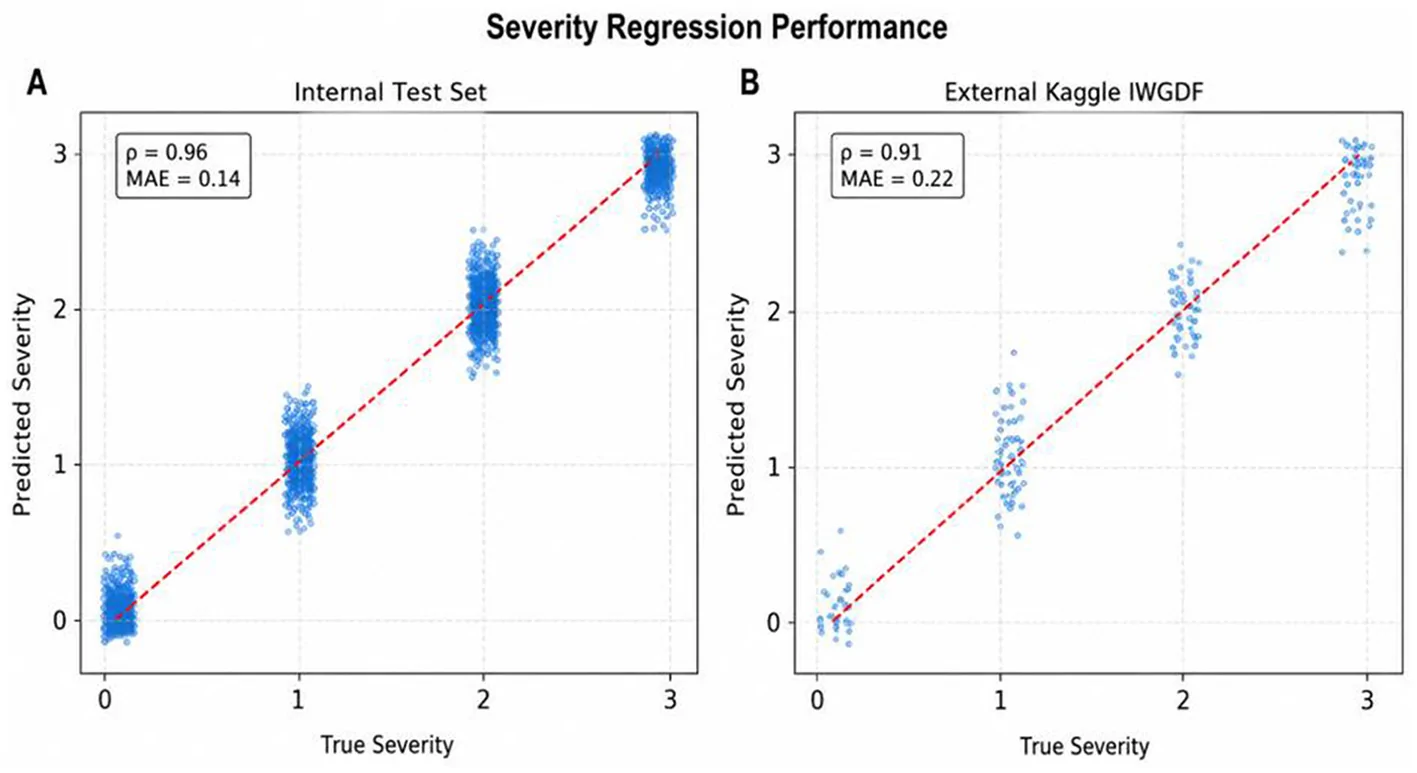
**(A)** Predicted versus clinician-assigned ordinal severity on the internal held-out test set. **(B)** Predicted versus clinician-assigned ordinal severity on the external Kaggle IWGDF validation set. Dashed lines indicate the fitted relationships; dispersion reflects residual error.

Severity-regression performance is reported in Table 10. Internal MAE was 0.14, and external MAE was 0.22 on the 0–3 ordinal scale. Spearman correlation remained high on the external set (ρ = 0.91), although the external error increase indicates a measurable domain shift. Figure 5 visualizes predicted vs. clinician-assigned scores and the fitted trend.

**Table 10:** Ordinal severity-regression performance on the internal held-out test set and the external ordinal validation set.

Evaluation set	MAE	RMSE	Spearman ρ
Internal held-out test set	0.14	0.29	0.96
External ordinal validation set	0.22	0.38	0.91

MAE and RMSE are on the 0–3 ordinal scale. Scores use the 0–3 ordinal target defined in Section 3.2.

### 4.8 Leave-one-source-out generalization

**Figure 6:**
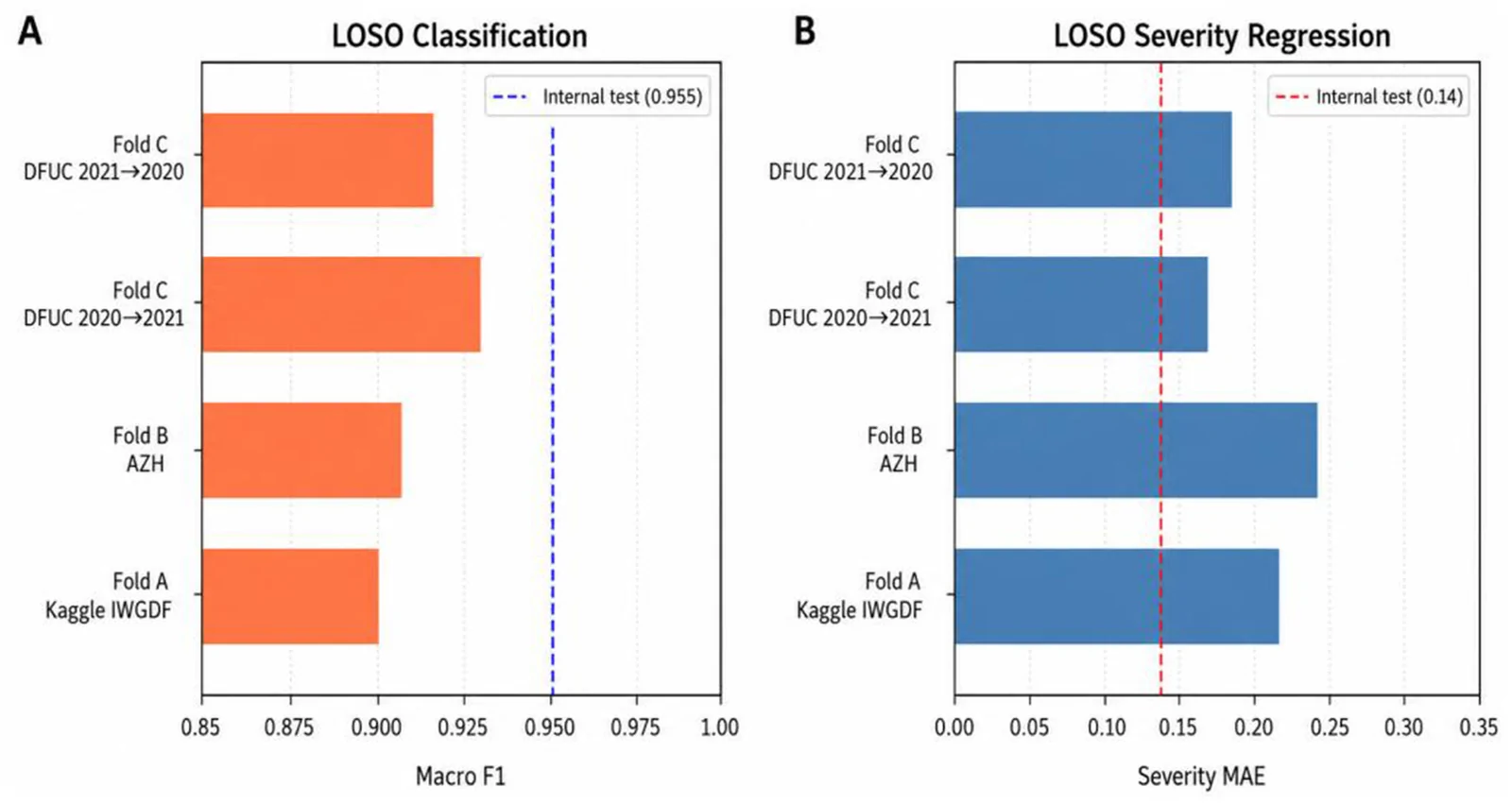
**(A)** LOSO classification performance, reported as macro F1 across the source-holdout folds. **(B)** LOSO severity-regression performance, reported as severity MAE across the source-holdout folds. Dashed vertical lines indicate the corresponding internal-test benchmarks.

LOSO results are shown in Table 11 and Figure 6. Macro F1 decreased relative to random-split evaluation, as expected under source shift. Performance was lowest on the externally held-out ordinal repository (macro F1 = 0.90; MAE = 0.22) and highest for the DFUC year-to-year transfer (macro F1 = 0.94).

**Table 11:** Leave-one-source-out generalization results.

Fold	Training sources	Held-out source	Macro F1	Severity MAE	Spearman ρ
A	DFUC 2020/2021 + AZH + Medetec	External ordinal repository	0.90	0.22	0.91
B	DFUC 2020/2021 + Medetec + eligible ordinal data	AZH	0.91	0.24	0.88
C	DFUC 2020	DFUC 2021	0.94	0.18	0.94
C reverse	DFUC 2021	DFUC 2020	0.93	0.19	0.93

Macro F1 is the unweighted mean of per-class F1 scores; severity MAE is mean absolute error on the 0–3 scale; Spearman ρ is rank correlation. Macro F1 is the unweighted mean of the seven per-class F1 scores computed from the held-out source images. Severity MAE is the mean absolute error on the 0–3 ordinal scale. Spearman ρ was calculated on the ordered pairs (predicted severity, clinician-assigned severity).

### 4.9 Prospective clinician-audited evaluation

**Figure 7:**
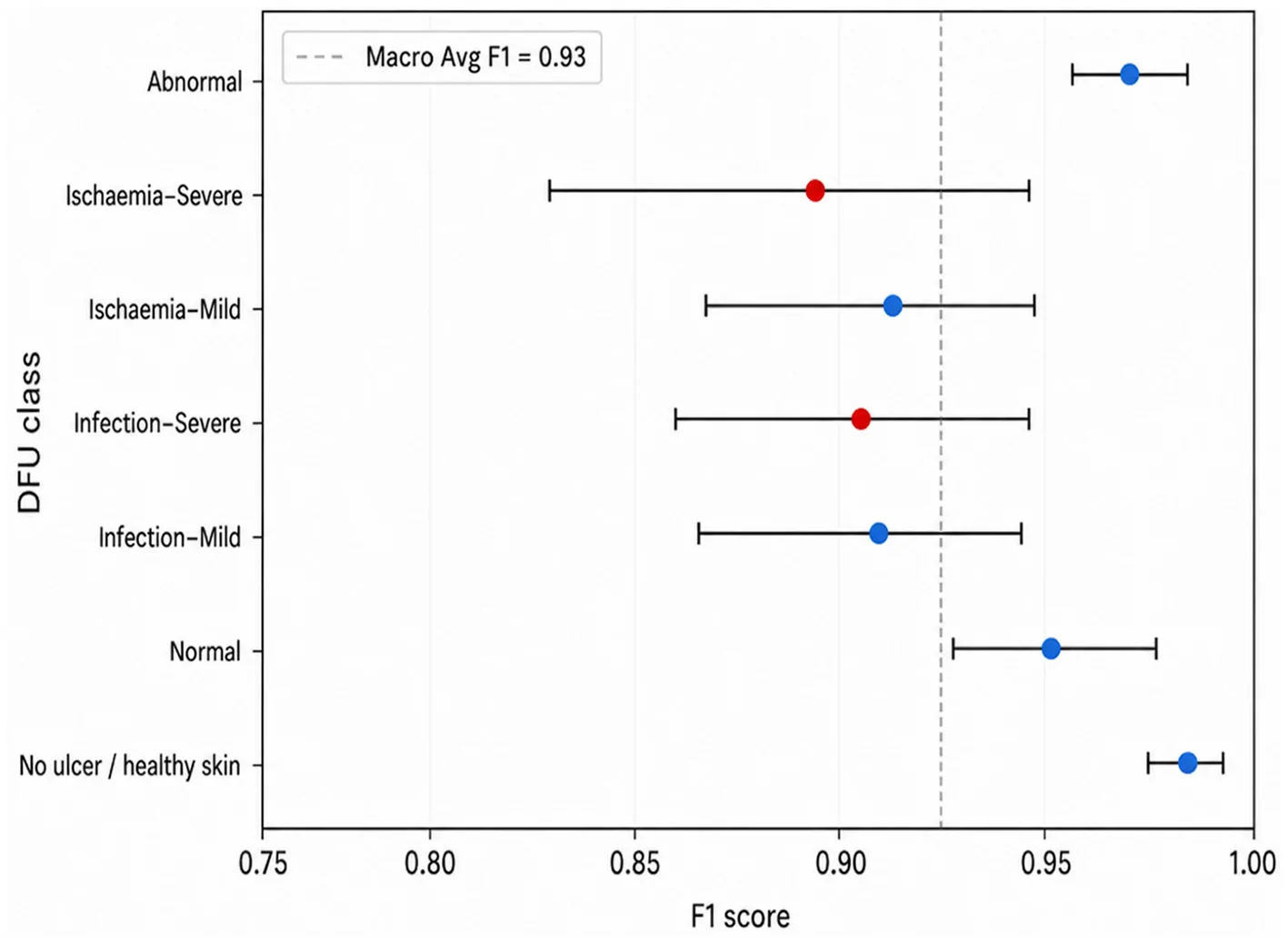
Class-specific sensitivity in the prospective clinician audit with 95% confidence intervals. The dashed reference line indicates the overall sensitivity level.

A total of 366 de-identified images were prospectively collected from patients and caregivers for the clinician-audited evaluation. Table 12 summarizes agreement, reviewer confidence, and recorded management-intent changes. Model output changed stated management intent in 101 cases (27.6%); this outcome should not be interpreted as proof of improved clinical outcomes. Figure 7 presents class-specific sensitivity estimates and confidence intervals.

**Table 12:** Summary of the prospective clinician-audited case study (366 real-world images).

Metric	Value
Images reviewed	366
Recorded management-intent changes after model review	101 (27.6%)
Explanations rated clinically useful	339 (92.6%)
High-confidence predictions (≥0.80)	342 (93.4%)
Cohen's κ, categorical model–clinician agreement	0.948
Weighted Cohen's κ, ordinal severity agreement	0.952
Inter-clinician Cohen's κ	0.935
Average clinician confidence	4.3/5
Sensitivity reduction under adverse imaging conditions	−4.8 percentage points

“Management-intent change” was prospectively recorded; it is not a patient-outcome endpoint. “Management-intent change” was prospectively recorded before and after display of the model output; it is not a patient-outcome endpoint.

Per-class clinician-audit metrics are shown in Table 13. Severe ischaemia had the lowest sensitivity (0.85), while healthy-skin cases had a sensitivity of 1.00. Macro F1 was 0.93 (95% bootstrap CI, 0.90–0.96).

**Table 13:** Class-wise performance in the 366-image clinician audit.

Class	Sensitivity	Specificity	Precision	F1	95% CI for F1	Mean confidence
No Ulcer/Healthy Foot Skin	1.00	0.99	0.98	0.99	0.97–1.00	0.97
Normal	0.94	0.98	0.96	0.95	0.92–0.98	0.93
Infection–Mild	0.91	0.96	0.91	0.91	0.87–0.95	0.89
Infection–Severe	0.88	0.98	0.93	0.90	0.85–0.94	0.91
Ischaemia–Mild	0.90	0.97	0.92	0.91	0.86–0.95	0.90
Ischaemia–Severe	0.85	0.99	0.94	0.89	0.82–0.94	0.92
Abnormal	0.98	0.98	0.97	0.97	0.95–0.99	0.95
Macro average	—	—	—	0.93	0.90–0.96	0.92
Micro average	—	—	—	0.94	0.92–0.96	0.92

Sensitivity, specificity, precision, and F1 were computed against the majority-vote clinician label; 95% bootstrap confidence intervals (1,000 resamples). Confidence intervals were obtained by 1,000-resample bootstrap.

### 4.10 Explainability and cross-branch consistency

Table 14 summarizes localization, concept sensitivity, and cross-branch attribution. Mean Grad-CAM–lesion IoU was 0.72 ± 0.11, attention-focus accuracy was 91%, and classification-vs.-severity attribution correlation was 0.84. TCAV associated visible bone/tendon exposure and necrosis most strongly with severe predictions. These results indicate association with clinically meaningful image concepts, not causal proof that the model reasons identically to a clinician.

**Table 14:** Explainability and attribution-consistency results.

Metric	Value	Interpretation/scope
Mean Grad-CAM/lesion-mask IoU	0.72 ± 0.11	Held-out explanation subset
Misclassified samples with boundary ambiguity	68%	Reviewer-coded
Global feature contribution	42%	SimCLR latent decomposition
Local feature contribution	58%	SimCLR latent decomposition
Attention-focus accuracy	91%	Attention overlapping lesion areas
TCAV: erythema	+0.39	Relative to Infection–Severe
TCAV: necrosis	+0.44	Relative to Ischaemia–Severe
TCAV: exudate	+0.31	Relative to infection classes
TCAV: visible bone/tendon	+0.51	Relative to Infection–Severe
Attribution consistency, classification vs. severity	0.84	Per-pixel correlation (SHAP/IG)

TCAV values are directional concept sensitivities (positive values indicate stronger association with the target class); they should not be interpreted as probabilities or causal effects. TCAV values are directional concept sensitivities; they should not be interpreted as probabilities or causal effects.

**Figure 8:**
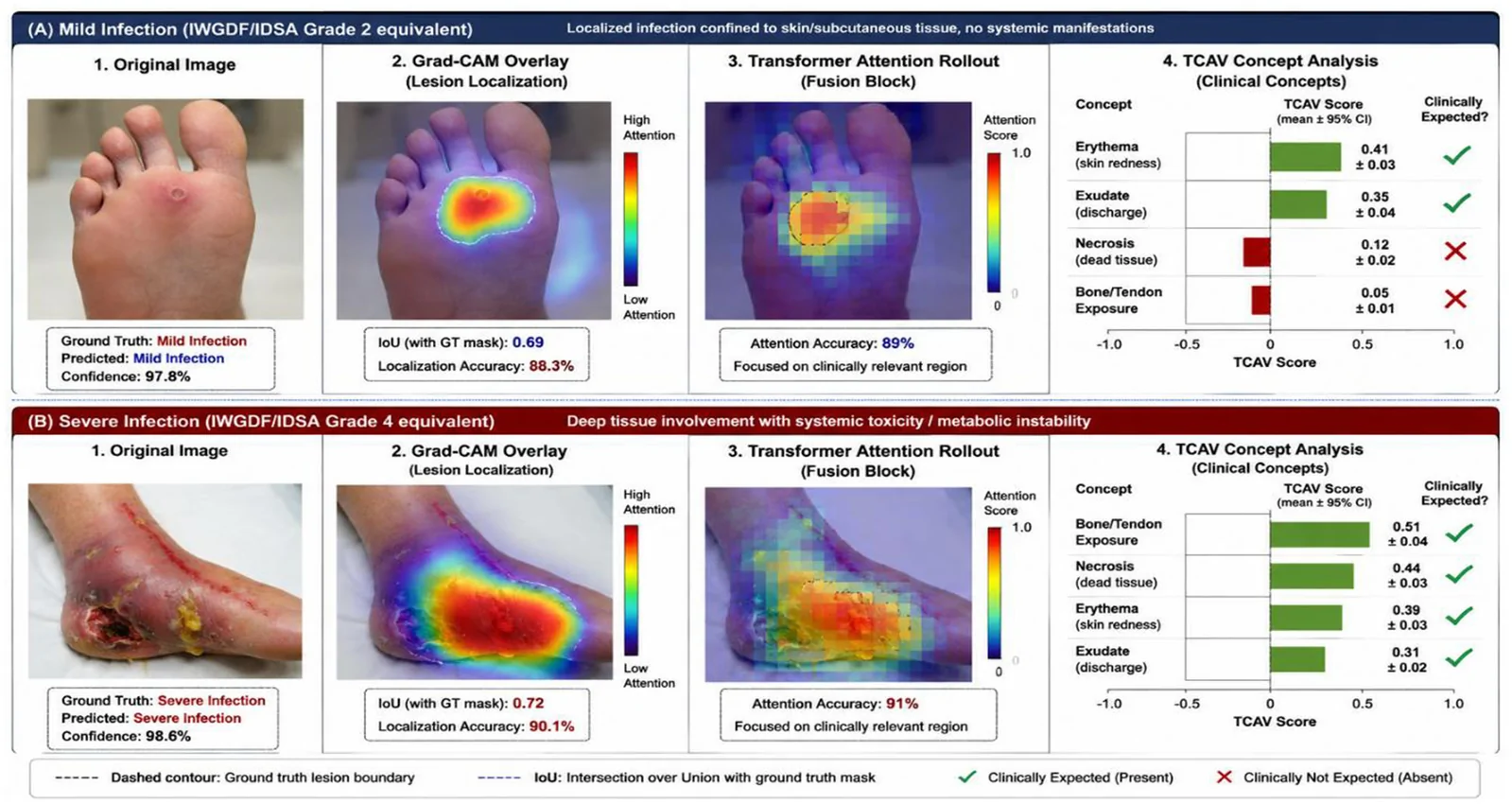
**(A)** Representative explanation for an Infection-Mild prediction and **(B)** representative explanation for an Infection-Severe prediction, combining the input image, Grad-CAM, transformer attention rollout, and TCAV concept scores.

**Figure 9:**
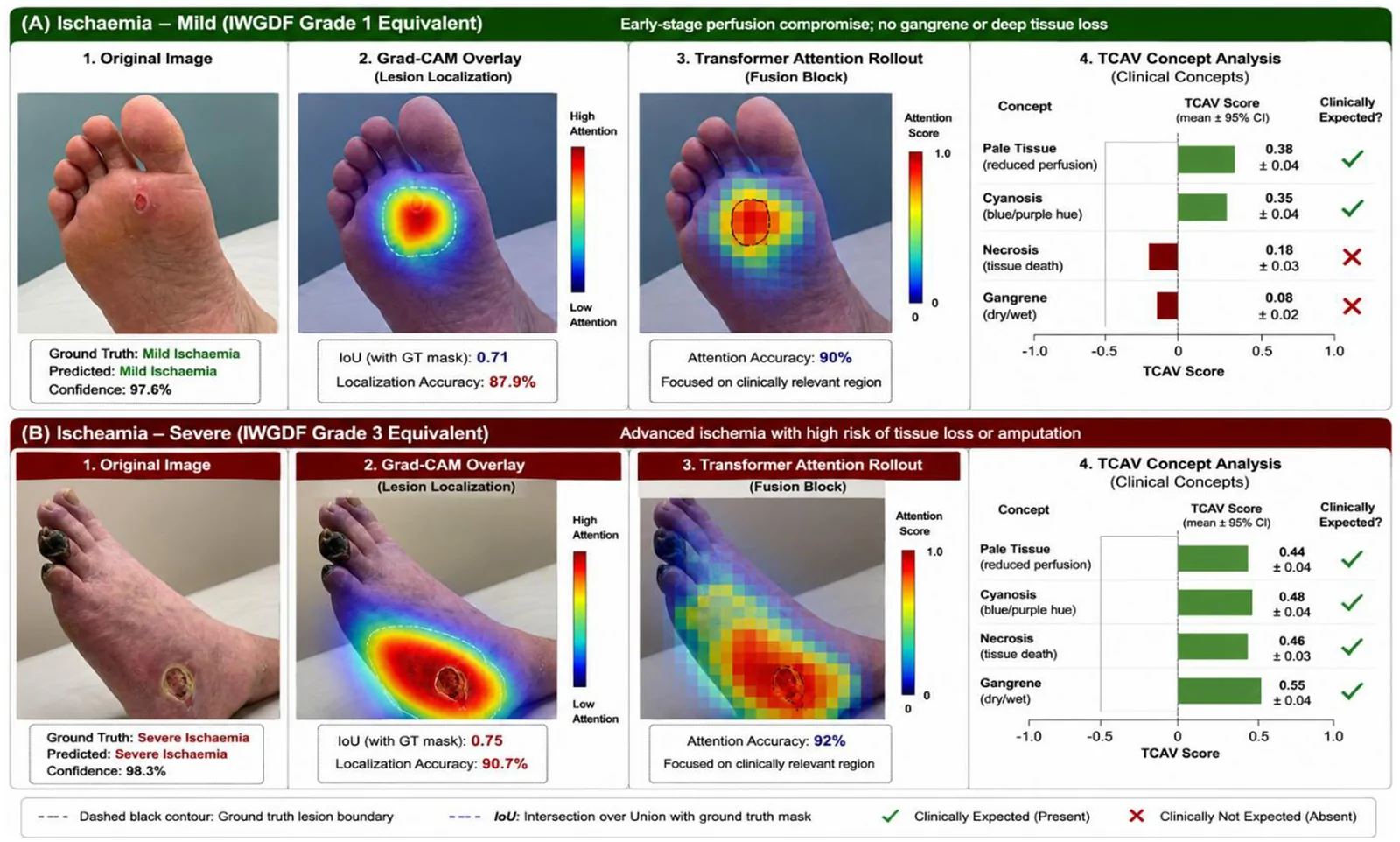
**(A)** Representative explanation for an Ischaemia-Mild prediction and **(B)** representative explanation for an Ischaemia-Severe prediction, combining spatial localization, transformer attention rollout, and concept-level evidence.

Figures 8, 9 provide representative multi-method explanations for severe infection and severe ischaemia. Grad-CAM localizes the lesion, attention rollout shows transformer focus, and TCAV ranks clinically defined concepts.

**Figure 10:**
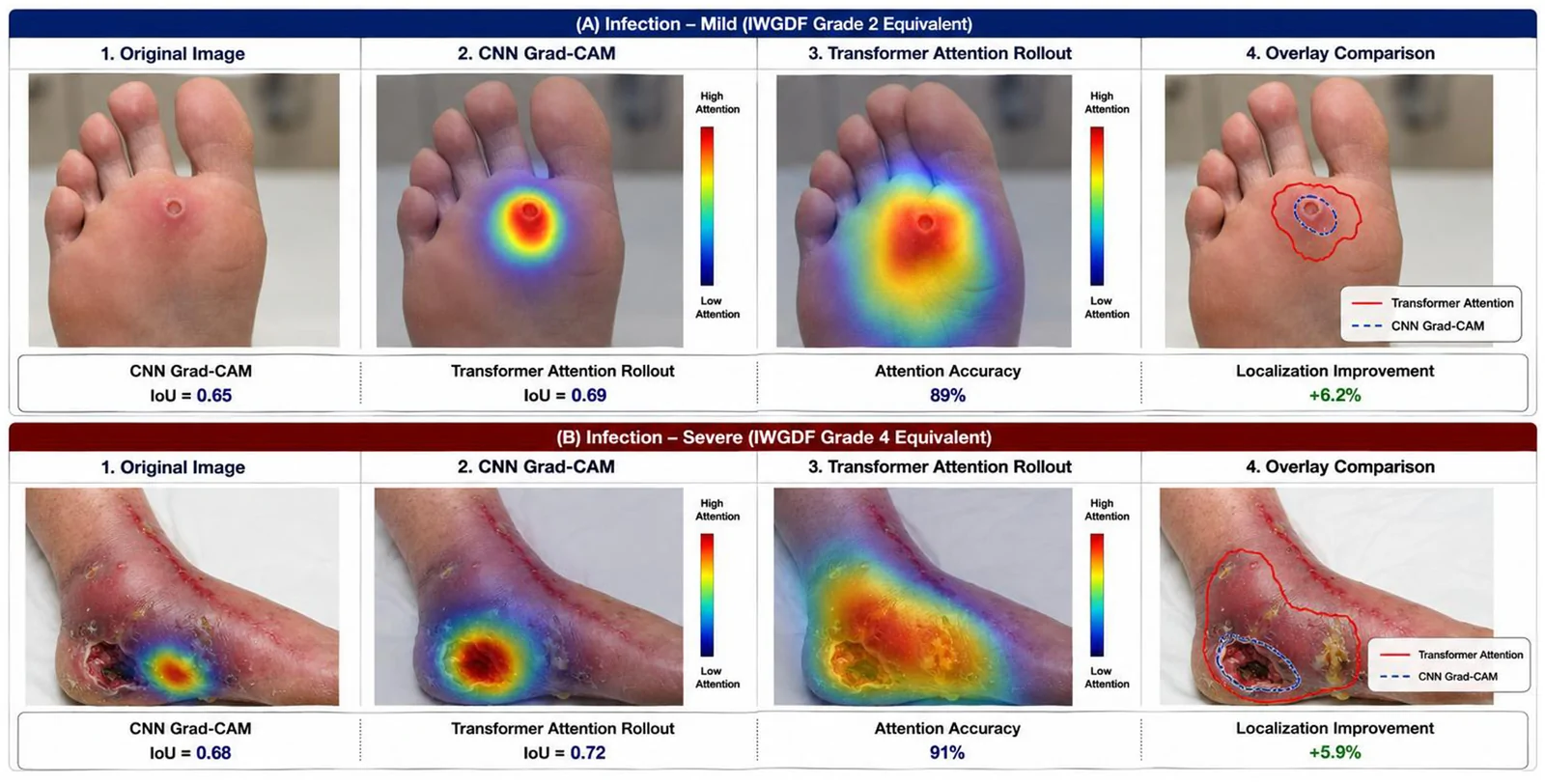
**(A)** Grad-CAM and transformer attention-rollout comparison for an Infection-Mild case. **(B)** Grad-CAM and transformer attention-rollout comparison for an Infection-Severe case.

**Figure 11:**
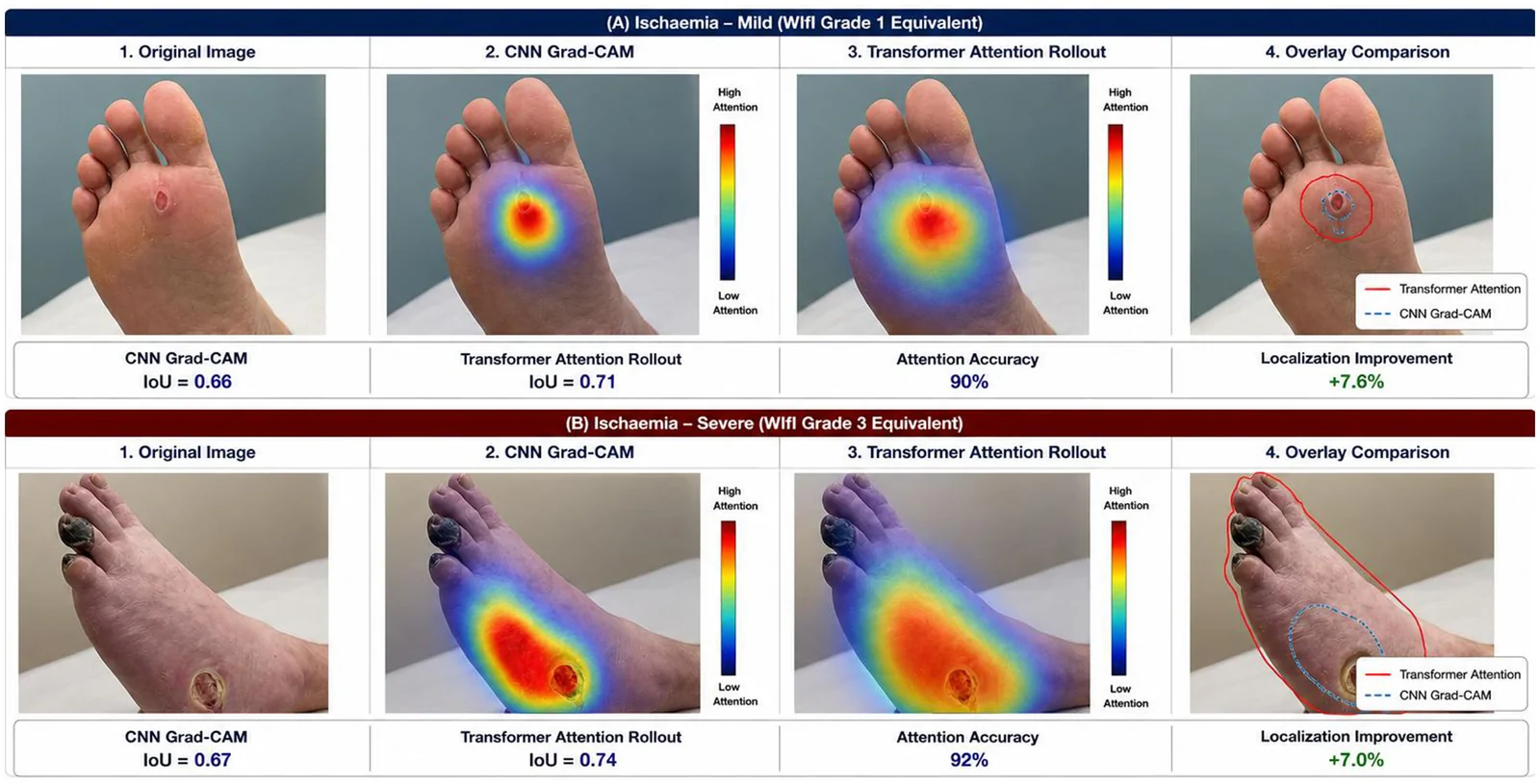
**(A)** Grad-CAM and transformer attention-rollout comparison for an Ischaemia-Mild case. **(B)** Grad-CAM and transformer attention-rollout comparison for an Ischaemia-Severe case.

Figures 10, 11 compare CNN Grad-CAM and transformer attention rollout directly. Their overlap supports cross-module consistency, while visible differences show that the two mechanisms are not redundant.

### 4.11 Interoperability and mobile deployment

Interoperability tests are summarized in Table 15. The DICOM/HL7 compliance rate was 98.7% across the tested exchange cases, not 100% of all clinical images. API call success was 99.9%, throughput was 47 images/s in the server test, and metadata traceability was complete for the audited pipeline. FHIR synchronization remained a controlled-test capability and was not part of the prospective workflow.

**Table 15:** Interoperability and systems-performance results.

Metric	Value	Scope
DICOM/HL7 exchange compliance	98.7%	150 synthetic and de-identified test cases
API call success	99.9%	Batch and single-case inference
Server streaming throughput	47 images/s	Controlled benchmark
Metadata traceability	100%	Preprocessing, augmentation, and acquisition logs

DICOM/HL7 compliance was tested on 150 synthetic and de-identified study cases; server throughput was measured under controlled benchmark conditions. FHIR synchronization was implemented and tested separately but was not enabled during the clinician audit.

**Figure 12:**
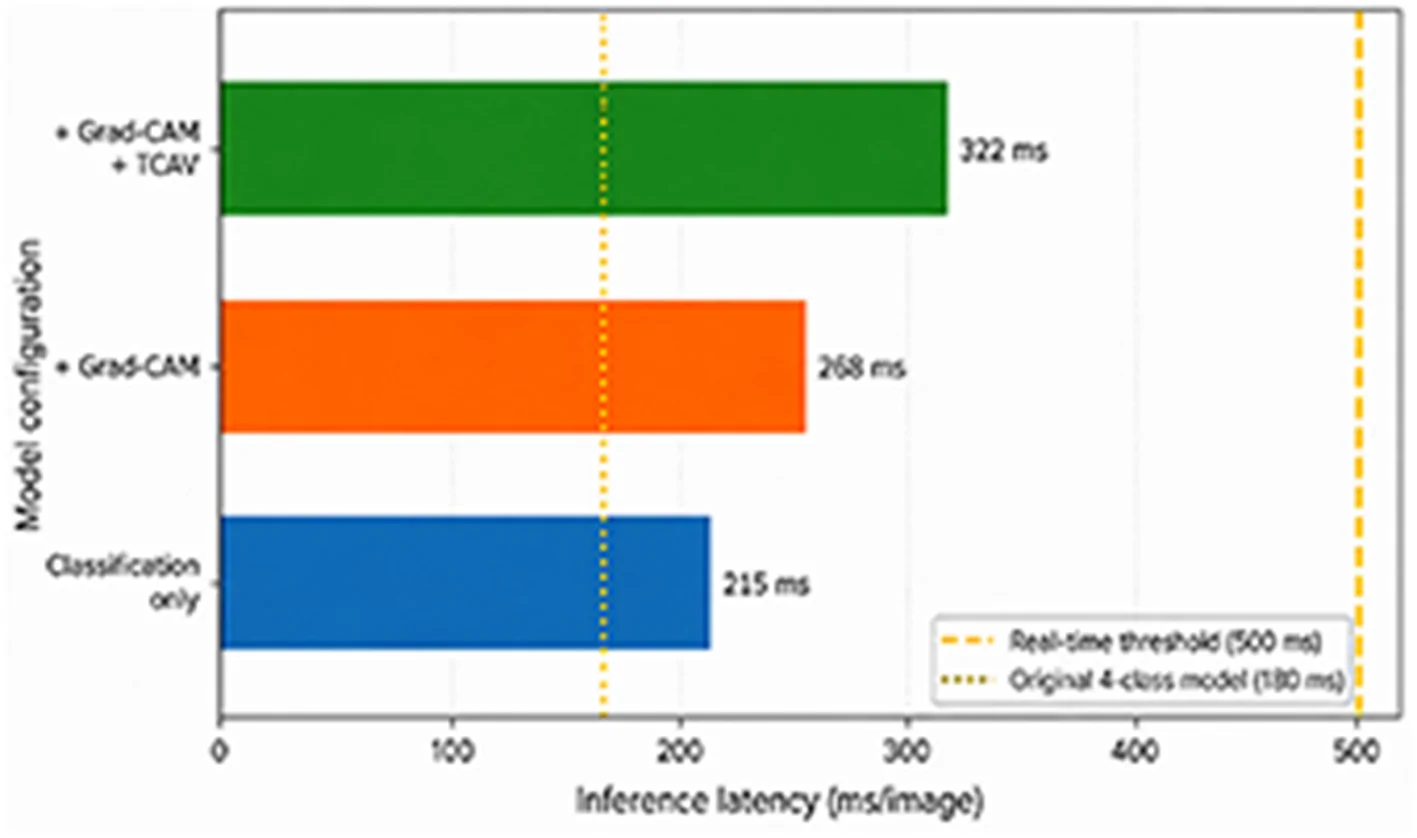
Mobile inference latency for classification only, classification plus Grad-CAM, and classification plus Grad-CAM and TCAV.

Table 16 and Figure 12 show mobile latency. Classification alone required 215 ms per image. Grad-CAM increased latency to 268 ms, and the full Grad-CAM + TCAV configuration required 322 ms. The 168 MB app footprint includes the quantized model and application assets; explainability did not require separate model files in the reported configuration.

**Table 16:** Mobile deployment profile on a Snapdragon 8 Gen 1 device (8 GB RAM).

Configuration	Latency (ms/image)	Frame rate (fps)	Quantized model (MB)	Total app footprint (MB)
Classification only	215	4.7	42	168
+ On-device Grad-CAM	268	3.7	—	—
+ On-device Grad-CAM and TCAV	322	3.1	—	—

Latency is the mean per-image inference time excluding camera capture and network transfer; frame rate = 1/latency. Frame rate is the reciprocal of mean latency and excludes camera capture and network transfer.

### 4.12 Qualitative class coverage and error examples

**Figure 13:**
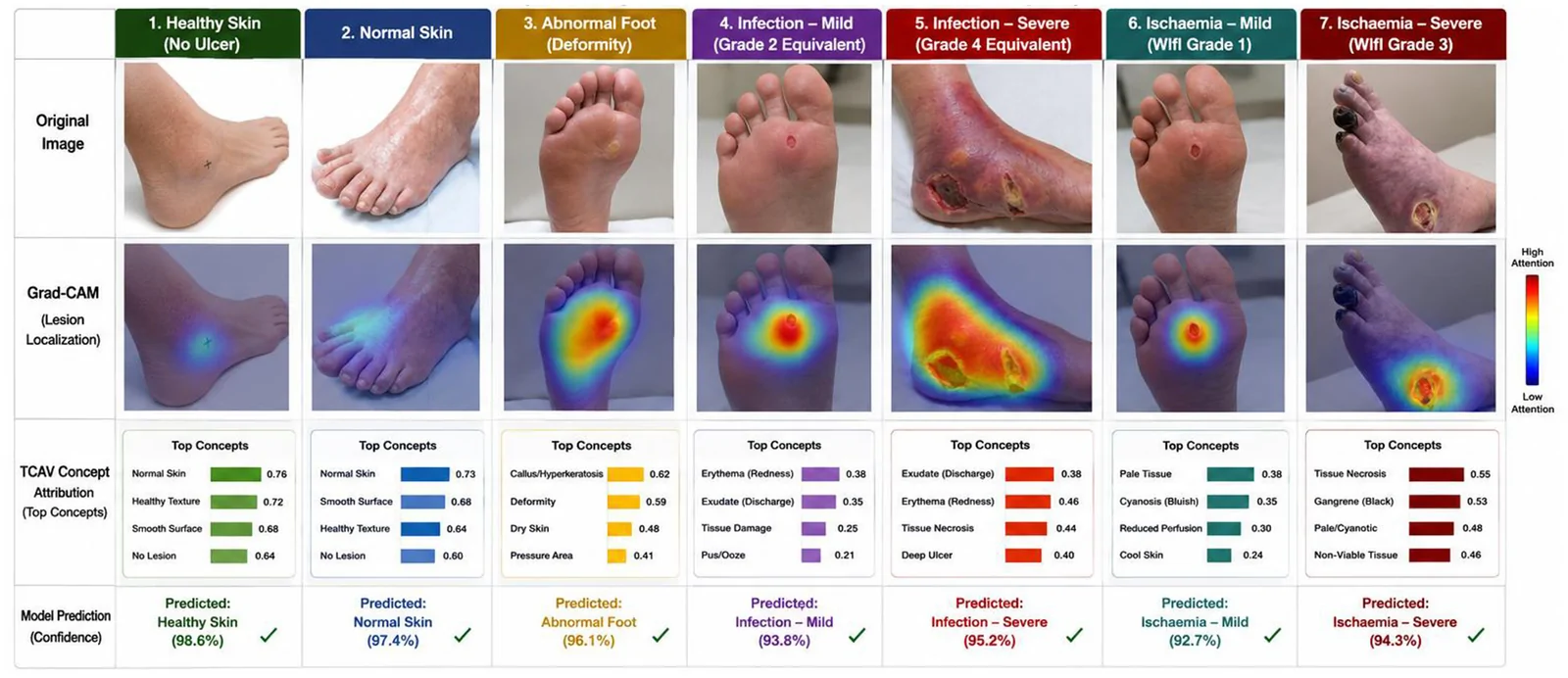
Representative examples from the seven output classes: healthy skin, Normal, Infection–Mild, Infection–Severe, Ischaemia–Mild, Ischaemia–Severe, and Abnormal.

**Figure 14:**
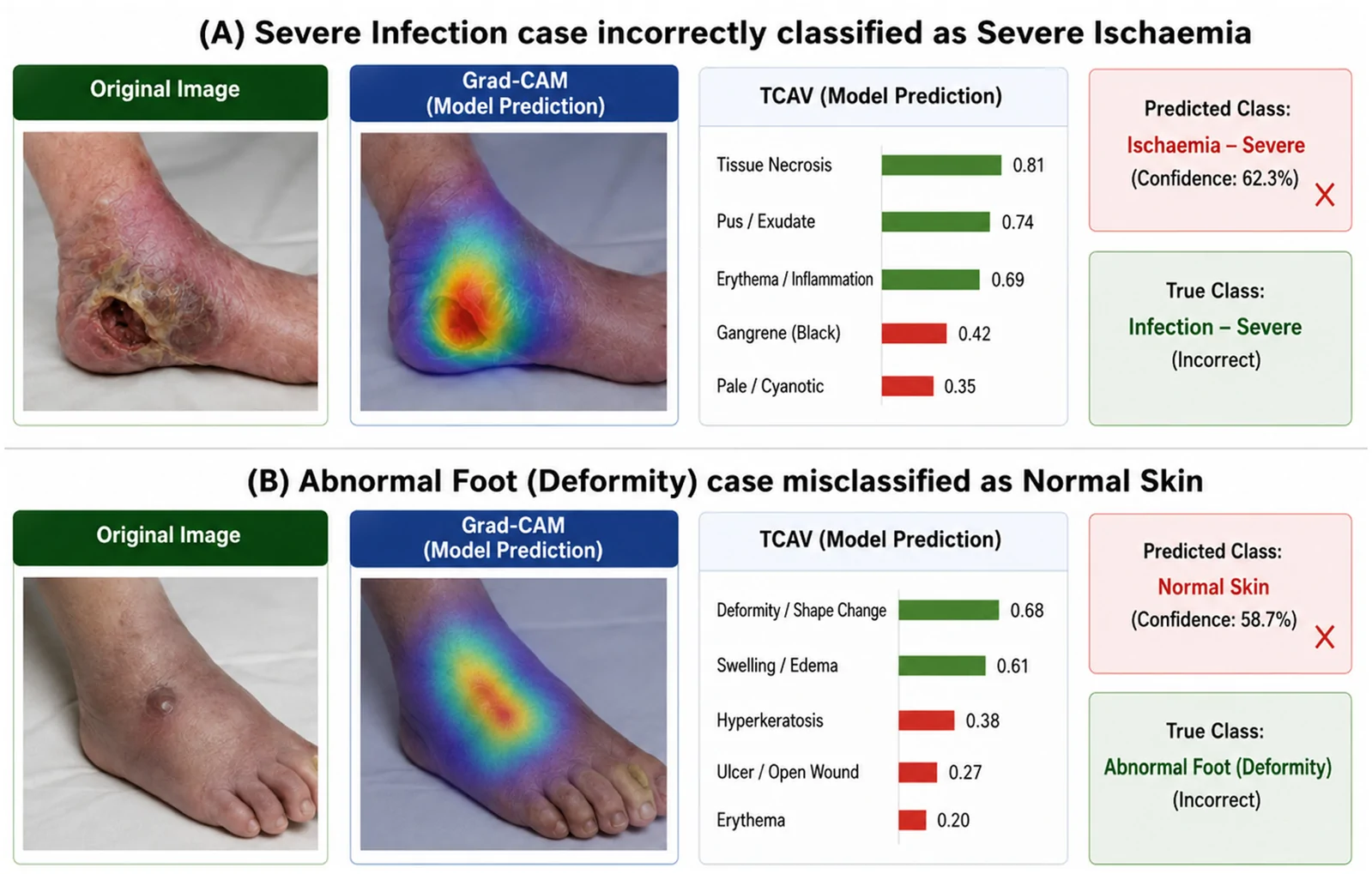
Representative misclassified cases: **(A)** Severe infection incorrectly classified as severe ischaemia; **(B)** Abnormal foot (deformity) misclassified as normal skin, with Grad-CAM localization and TCAV concept attribution.

Figure 13 provides representative images across all seven classes and illustrates the intended visual taxonomy. Figure 14 shows difficult or misclassified cases, including adjacent-severity ambiguity, low contrast, illumination variation, and incomplete wound framing. These examples contextualize the remaining severe-class false negatives and support the need for quality control and clinician oversight.

## 5 Discussion and analysis

The proposed framework is a seven-class, severity-aware, multi-task diagnostic system for DFU classification. The corrected held-out endpoint was 95.5% accuracy with macro F1 0.953. Five-fold cross-validation remained stable at 96.9% ± 0.2 percentage points, and ablation indicated incremental validation gains from SimCLR, U-Net refinement, transformer fusion, and severity supervision. Importantly, the paper now distinguishes original-corpus size, augmented training exposure, validation accuracy, held-out accuracy, and external performance rather than presenting them as interchangeable quantities.

LOSO macro F1 of 0.90–0.94 demonstrates useful but imperfect cross-source transfer. The external ordinal repository produced higher MAE than the internal test set (0.22 vs. 0.14), confirming that source and annotation shift remain relevant. The clinician audit provided an additional real-world check, with macro F1 0.93 and weighted κ 0.952. The 27.6% management-intent-change rate suggests that model output can affect clinician thinking, but it does not establish improved treatment or outcomes. A controlled clinical-impact study would be required for that claim.

The most consequential residual errors involved adjacent severe classes. FNRs were 8.9% for Infection–Severe and 10.3% for Ischaemia–Severe. Although both were below the internal 15% engineering screen, a false reassurance risk remains. The severity branch can flag some adjacent-class errors through an elevated continuous score, but it cannot guarantee safe triage. Deployment should therefore include image-quality checks, uncertainty display, conservative referral rules, and explicit labeling as decision support rather than autonomous diagnosis.

Spatial localization, concept sensitivity, attention rollout, and cross-branch attribution were mutually supportive. TCAV's strongest severe-class concepts—visible bone/tendon and necrosis—were clinically plausible, and 92.6% of clinician reviews rated the explanations useful. However, explanation agreement is not proof of causal faithfulness. Concept sets may omit relevant cues, saliency can be unstable, and clinician usefulness ratings may be influenced by the model's predicted class. Future evaluation should include randomized explanation conditions and counterfactual tests.

The transformer and multi-task head increased model complexity, while on-device explanation added a further 107 ms relative to classification alone. The resulting 322 ms mean latency met the project's 500 ms engineering target on the reference device. This threshold is a usability target, not a universal clinical standard. Lower-end hardware, thermal throttling, camera processing, and full application workflows may produce longer delays. The three-configuration profile allows implementers to choose between faster classification and richer explanations.

### 5.1 Comparison with prior work

Table 17 compares the present framework with representative, verifiable DFU studies. Earlier works established DFU classification, infection/ischaemia recognition, segmentation, transformer fusion, few-shot severity grouping, explainability, and mobile deployment as separate capabilities. The present contribution is the integrated evaluation of a multi-source seven-class taxonomy, ordinal severity regression, source-holdout generalization, concept-based explanations, clinician agreement, and explanation-inclusive mobile latency. The table avoids unsupported “first-ever” language and describes the documented feature set instead.

**Table 17:** Comparison of the proposed framework with representative DFU image-analysis studies.

References	Sources	Classes/severity	Self-supervised	Multi-task severity	Explainability	External generalization	Clinician audit	Mobile profiling
Karthik et al. (2025)	DFUC 2021	Four-class DFUC	No	No	Grad-CAM	Internal	No	No
Yap et al. (2021)	Small DFU severity datasets	Zero/mild/severe	No	Severity classification	Not central	Internal	No	Low-resource focus
Sait and Nagaraj (2025)	DFUC 2021	Four-class DFUC	No	No	SHAP	Internal	No	No
Goyal et al. (2018)	Single DFU collection	Binary DFU/healthy	No	No	None	Cross-validation	No	No
Goyal et al. (2020)	DFUC pathology dataset	Binary infection and ischaemia tasks	No	No	None	Internal	No	No
Proposed framework	DFUC 2020/2021 + AZH + Medetec + ordinal repository	Seven classes + 0–3 severity	SimCLR	Classification + regression	Grad-CAM, TCAV, SHAP/IG, rollout	Random split + LOSO	Categorical + ordinal agreement	Class-only, Grad-CAM, TCAV

“Yes” indicates the feature is reported; “NO” indicates not evaluated.

### 5.2 Limitations and future work

First, several sources lacked patient identifiers, so perceptual hashing could not exclude all same-patient images acquired from different angles. Second, severity labels transferred onto DFUC images were clinician-supervised image labels rather than full bedside grades. Third, the clinician audit was single-site and modest in size. Fourth, the healthy-skin class had only 346 original images and 52 held-out examples, despite augmentation in training; its near-perfect recall therefore has a wider practical uncertainty than the point estimate suggests. Fifth, the study evaluates images rather than multimodal clinical records, longitudinal wound trajectories, or patient outcomes. Sixth, the source record did not include raw fold-level difference vectors, so effect-size estimates were not reconstructed. Finally, mobile profiling was performed on one device class.

Future work should prioritize multicenter prospective validation, demographic and skin-tone subgroup analysis, externally adjudicated severity labels, temporal modeling, fusion with perfusion and clinical variables, lower-resource-device profiling, and preregistered clinical-impact endpoints. Explanation evaluation should include stability, counterfactual faithfulness, and blinded clinician studies. Code, split manifests, label mappings, pHash clusters, and inference configuration should be released with persistent identifiers to support independent replication.

## 6 Conclusion

This paper presented a seven-class, severity-aware, multi-task CNN–Transformer framework for diabetic foot ulcer classification and severity assessment. Images from DFUC 2020/2021, AZH, Medetec, and an IWGDF-aligned ordinal repository were harmonized into a clinically meaningful taxonomy comprising healthy skin, uncomplicated and abnormal ulcer presentations, and mild and severe infection and ischaemia. Explicit provenance tracking, patient- or source-aware partitioning, perceptual-hash deduplication, and post-split augmentation were used to reduce data leakage and improve reproducibility. The proposed architecture integrates domain-adaptive SimCLR pretraining, U-Net-based image refinement, EfficientNet-B0 feature extraction, lightweight Swin-style transformer fusion, and parallel classification and ordinal-severity heads. On the independent held-out test set, the framework achieved 95.5% accuracy and a macro F1-score of 0.953, while five-fold cross-validation on the training+validation partition produced a mean accuracy of 96.5% ± 0.3 percentage points. Ablation experiments demonstrated that each architectural component contributed incrementally to performance, with the complete model attaining 97.2% validation accuracy. Leave-one-source-out evaluation further showed meaningful, although imperfect, cross-source generalization, with macro F1-scores ranging from 0.90 to 0.94. In the prospective 366-image clinician audit, the model achieved a weighted Cohen's κ of 0.952 for severity agreement. Model-assisted review changed recorded clinical management intent in 27.6% of cases, while 92.6% of explanations were judged clinically useful. Full on-device classification with Grad-CAM and TCAV required 322 ms per image, supporting point-of-care feasibility. Nevertheless, the framework should be regarded as interpretable clinical decision support rather than autonomous diagnosis. Future research should prioritize multicentre prospective validation, longitudinal outcome assessment, broader demographic and device-level evaluation, and continued reduction of severe-class false negatives.

## Data Availability

The datasets analyzed in this study were obtained from existing repositories and are subject to the access, licensing, and data-use conditions specified by their respective providers. The DFUC 2020 and DFUC 2021 datasets comprise de-identified high-resolution diabetic foot ulcer images with expert annotations and are available only in accordance with the usage agreements and access requirements established by the original dataset providers. Requests for access to these datasets should be directed through the official DFUC websites (https://dfu-challenge.github.io/dfuc2020.html and https://dfu-challenge.github.io/dfuc2021.html) or to Professor Moi Hoon Yap, Manchester Metropolitan University (m.yap@mmu.ac.uk). The AZH Wound and Vascular Center dataset, Medetec dataset, and IWGDF-aligned ordinal repository are available from their respective original repositories subject to the applicable access, licensing, ethical, and data-use terms. The prospectively collected, clinician-audited case-study images generated as part of this study are not publicly available because of participant privacy, consent, and institutional requirements. Where permitted, de-identified summary data associated with the prospective case study may be made available by the corresponding author upon reasonable request and subject to institutional approval and applicable ethical and privacy restrictions.
