# Supplementary material for: A severity-aware multi-task CNN-transformer framework for explainable diabetic foot ulcer triage and mobile deployment
Source: Front Artif Intell. 2026 Sep 3;9:1795751. doi: 10.3389/frai.2026.1795751 (PMC13582628; doi:10.3389/frai.2026.1795751)
Supplement: Supplementary file 1 [file Table_1.docx]

**SUPPLEMENTARY MATERIAL**

**Training Configuration, Hyperparameters, and Model Weights**

This supplementary file summarizes the principal training settings, selected hyperparameters, and model artifacts reported for the proposed diabetic foot ulcer classification and severity-estimation framework.

**S1. Training Setup**

| **Item** | **Configuration** |
| --- | --- |
| Software | Python 3.10; TensorFlow 2.12; Keras |
| Hardware | NVIDIA A100 GPU (40 GB) |
| Input image size | 224 × 224 × 3 |
| Training stages | SimCLR pretraining → U-Net refiner pretraining → supervised multi-task fine-tuning |
| Training / validation / held-out test split | 19,940 / 1,246 / 3,739 original images |
| Effective augmented training set | 42,000 images |
| Reproducibility | NumPy, TensorFlow, and Python random seeds fixed; configurations recorded in YAML |

**S2. SimCLR Pretraining**

| **Parameter** | **Selected setting** |
| --- | --- |
| Backbone | EfficientNet-B0 |
| Epochs | 100 |
| Batch size | 256 |
| Projection head | Two-layer MLP; hidden 128; output 128; L2 normalization |
| Temperature | 0.1 |
| Loss | NT-Xent |
| Optimizer | Adam |
| Initial learning rate | 0.001 |
| Learning-rate schedule | Cosine decay without restarts |
| Weight decay | None |
| Main augmentations | Random resized crop, horizontal flip, color jitter, grayscale, Gaussian blur |

**S3. U-Net Refiner Pretraining**

| **Parameter** | **Selected setting** |
| --- | --- |
| Architecture | Encoder filters [64, 128] with symmetric decoder |
| Activation | ReLU; tanh output |
| Loss | Combined SSIM + L1 |
| Optimizer | Adam |
| Learning rate | 1 × 10⁻⁴ |
| Batch size | 32 |
| Maximum epochs | 50 |
| Early stopping | Validation loss; patience 5; minimum delta 1 × 10⁻⁴ |

**S4. Supervised Multi-Task Fine-Tuning**

| **Parameter** | **Selected setting** |
| --- | --- |
| Optimizer | AdamW (β₁ = 0.9, β₂ = 0.999, ε = 1 × 10⁻⁷) |
| Weight decay | 1 × 10⁻³ |
| Batch size | 32 |
| Learning-rate schedule | 7 × 10⁻⁴ for first 25 epochs; 3 × 10⁻⁴ for next 15; 1 × 10⁻⁴ thereafter |
| ReduceLROnPlateau | Factor 0.5; patience 3; minimum LR 1 × 10⁻⁶ |
| Early stopping | Patience 8 on validation loss |
| Classification loss | Focal loss; γ = 1.0; α = 0.5 |
| Severity loss | Huber loss; δ = 1.0 |
| Severity task weight λ | 0.6 |
| Label smoothing | 0.1 |
| Classification dropout | 0.4 after Dense(512); 0.3 after Dense(256) |
| Severity dropout | 0.3 after Dense(128) |
| Transformer | 2 attention heads; 1 transformer layer |

**S5. Hyperparameter Search**

| **Hyperparameter** | **Search space** | **Selected** |
| --- | --- | --- |
| Initial learning rate | {7×10⁻⁴, 5×10⁻⁴, 3×10⁻⁴, 1×10⁻⁴} | 7×10⁻⁴ |
| Weight decay | {1×10⁻³, 1×10⁻⁴, 1×10⁻⁵} | 1×10⁻³ |
| Batch size | {16, 32, 64} | 32 |
| Classification dropout | {0.2, 0.3, 0.4, 0.5} | 0.4 / 0.3 |
| Severity dropout | {0.2, 0.3, 0.4} | 0.3 |
| Label smoothing | {0, 0.05, 0.1, 0.15, 0.2} | 0.1 |
| Transformer heads | {2, 4} | 2 |
| Transformer layers | {1, 2} | 1 |
| Severity weight λ | {0.2, 0.4, 0.6, 0.8, 1.0} | 0.6 |

**S6. Final Model and Training Summary**

| **Item** | **Reported value** |
| --- | --- |
| Final retraining set | Combined training + validation set: 21,186 images |
| Internal early-stopping split | Random 10% of the combined training + validation set |
| Full model trainable parameters | 6.4 million |
| Full model training time | 10.3 hours |
| Validation accuracy | 97.2% |
| Validation macro F1 | 0.955 |
| Severity MAE | 0.14 |
| Held-out test accuracy | 95.5% |
| Held-out macro F1 | 0.953 |

**S7. Model Artifacts**

| **Artifact** | **Description** |
| --- | --- |
| SimCLR backbone weights | EfficientNet-B0 weights obtained after contrastive pretraining |
| U-Net refiner weights | Best refiner model selected using validation loss |
| Final multi-task model weights | Best checkpoint selected using validation macro F1 and severity MAE |
| Mobile model | TensorFlow Lite model using dynamic/float16 quantization and pruning; reported model size 42 MB |
| Training logs | Training-history records for the SimCLR, U-Net, and supervised multi-task stages |
| Hyperparameter configuration | YAML configuration containing the selected training settings |

*^*^The above items constitute the core supplementary information supporting the manuscript statement that training logs, hyperparameter configurations, and final model weights are provided as supplementary material.*
